# Experiences and challenges of family caregivers of people with lower limb amputation: a qualitative study

**DOI:** 10.1590/1980-220X-REEUSP-2024-0264en

**Published:** 2025-05-26

**Authors:** Diana Fonseca Rodrigues, António Luís Rodrigues Faria de Carvalho, Cristina Maria Correia Barroso Pinto

**Affiliations:** 1 Universidade do Porto, Faculdade de Medicina, RISE-Health, Porto, Portugal.; 2 Universidade do Porto, Instituto de Ciências Biomédicas Abel Salazar, Porto, Portugal.; 3 Escola Superior de Enfermagem do Porto, Porto, Portugal.

**Keywords:** Amputees, Empowerment, Caregivers, Hospital to Home Transition, Lower Extremity, Qualitative Research

## Abstract

**Objective:**

To explore the experiences of family caregivers transitioning to their roles of caregivers for people with dysvascular major lower limb amputation, identify the challenges they face, understand their specific needs during this transition, examine their experiences during the hospital-to-home transition, and gather insights into strategies that promote empowerment in caregiving.

**Method:**

This exploratory, cross-sectional descriptive study employed a qualitative approach, conducting semi-structured interviews with 40 family caregivers of individuals with dysvascular major lower limb amputation. Data were analyzed using ATLAS.ti software and Bardin’s content analysis method.

**Results:**

Content analysis identified five categories: 1) family caregiver role, 2) amputee needs/difficulties, 3) family caregiver needs/difficulties, 4) home transition, and 5) strategies to promote family caregiver empowerment, highlighting critical aspects of the caregiving experience and identifying areas for intervention.

**Conclusion:**

The findings emphasize the need for family-centered empowerment programs combining hospital-based training with community support. Future research should evaluate their impact on reducing caregiver burden and improving outcomes for both caregivers and amputees.

## INTRODUCTION

Limb amputation poses a significant challenge to global health, profoundly affecting the lives of individuals and their families. Dysvascular conditions, primarily caused by peripheral arterial disease (PAD) and diabetes mellitus (DM), are the leading causes of lower extremity amputations^(1,2)^. Over 80% of lower extremity amputations are due to dysvascular causes, primarily peripheral arterial disease (PAD), diabetes mellitus (DM), or a combination of both^(3)^. Studies measuring functional capacity 12 months after dysvascular lower limb amputation (LLA) have reported poor physical function, with only 39% of patients returning to their previous level of mobility^(4)^. Undergoing lower limb amputation is a life-altering event that can negatively impact both physical and mental health^(2)^.

Rehabilitation outcomes following dysvascular amputation are often poor, with affected individuals experiencing a level of disability greater than 95% in relation to the general population^(5)^. Lower limb amputations (LLA) can result in physical limitations that may restrict amputees’ functional abilities in daily activities, often leading to a significant loss of independence and increased dependence on others^(1)^. A dependent person is someone who has limited capacity or is unable to start and carry out activities essential for well-being, health, and maintaining life without assistance from another person^(6)^. Individuals may experience situations in which they cannot meet their self-care needs sufficiently, resulting in self-care deficits. This can be caused by factors such as illness, injury, disability, or lack of knowledge and resources^(7)^.

Informal caregivers play a crucial role in supporting relatives by assisting with ADLs, performing medical and nursing tasks, providing psychosocial support, and communicating with healthcare professionals^(8)^. Family caregivers are relatives, friends, partners, or neighbors who offer unpaid support to individuals with physical, mental, or cognitive limitations. The competence, skills, and motivation of caregivers may vary due to several reasons^(9)^. Previous studies have shown that amputation care creates psychological, financial, and physical stress in families. The caregiver burden increases significantly with the severity of the amputation, with major amputations placing heavier strain than minor ones^(10)^. Caregiver tasks progressively increase in intensity as the care recipient’s disability worsens, becoming more time-consuming, complex, and stressful. These growing demands can lead to negative emotional responses, which may, in turn, trigger behavioral or physical reactions, increasing the caregiver’s risk for mental or physical health issues^(9)^.

A change in the health condition of a family member will cause other family members to take on the role of caregiver, besides triggering a transition within the family. A family member who is willing to take the responsibility of a caregiver will undergo a situational transition, which will bring about changes in both their own life and the life of the dependent person. Learning and acquiring new skills are the essential components to this process^(11)^. Supporting informal caregivers is essential not only for their well-being but also for society. Highlighting the heavy burden and often unmet needs of caregivers can help the healthcare community to become aware regarding the impact of amputation on a patient’s family^(10)^.

Family caregivers play a critical role in the care plans for patients with chronic illnesses. As patient dependence on caregivers grows, nurses face challenges in ensuring both patient safety and quality of care. By strengthening communication and providing robust support, nurses can enhance caregivers’ competence by equipping them with the skills required to maintain patient safety^(12)^. Nurses can implement interventions to empower them to mitigate the burden on family caregivers. Unlike traditional education models, caregiver empowerment emphasizes self-management, transforming passive information delivery into active collaboration between caregivers and patients^(13)^.

This study is part of a broader doctoral research project exploring the perspectives of nurses and dysvascular major lower limb amputees. It aims to contribute to the development of interventions and programs to support family caregivers in managing the care process and addressing the physical, emotional, and social dimensions of caregiving. The main objectives of this study were as follows:

- To explore the experience of transitioning into the role of family caregiver for a person with dysvascular major lower limb amputation.
- To identify the difficulties and challenges faced by family caregivers in caring for a person with dysvascular major lower limb amputation.
- To identify the needs/difficulties of family caregivers of a person with dysvascular major lower limb amputation in their transition to the caregiver’s role.
- To explore the experience of family caregivers of a person with dysvascular major lower limb amputation during the hospital/home transition.
- To understand the perspectives of family caregivers regarding strategies to promote their empowerment in caring for a person with dysvascular major lower limb amputation.

## METHOD

### Study Design and Settings

This was an exploratory, cross-sectional, descriptive, and qualitative study conducted in a vascular surgery unit in a hospital in Northern Portugal. During the study, we carefully considered rigorous research criteria and followed the Consolidated Criteria for Reporting Qualitative Studies (COREQ) for reporting qualitative research. Data were collected between May 2022 and June 2023 by one of the researchers with experience in conducting interviews who was not a member of the unit’s team.

### Selection of Participants

The participants were selected from family caregivers who accompanied patients with dysvascular major lower limb amputation to the follow-up hemodynamic consultation for vascular diseases in a Vascular Surgery Service in a hospital in Northern Portugal.

Family caregivers were recruited according to the following criteria: (1) being 18 years or older; (2) providing care to a dysvascular major lower limb amputee at home; (3) being identified as primary caregivers who had been taking care of the amputee since discharge; and (4) caring for amputees who required assistance with activities of daily living.

Data collection was conducted for over a 13-month period, targeting the entire population of family caregivers of patients with major lower limb amputations due to peripheral artery disease (PAD) who attended post-amputation hemodynamic follow-up consultations for vascular diseases. The selection of participants was based on achieving data saturation, which occurred after conducting 40 interviews with the family caregivers of dysvascular amputees. Saturation is defined as the point at which additional data can no longer contribute to new insights for the phenomenon under study, leading to redundancy. According to Bardin^(14)^, researchers must be vigilant during categorization and analysis to recognize when data stop providing new elements, marking the attainment of saturation, which was confirmed in this study after 40 interviews.

In this qualitative study, potential biases were identified and steps taken to mitigate them. One of the main concerns was selection bias, as participants may have been selected based on their availability or willingness to participate. To minimize this bias, efforts were made to include individuals from different age groups and socioeconomic backgrounds. Although recruitment was not conducted across multiple contexts, this approach helped to ensure a more varied and representative sample of these family caregivers.

### Data Collection

Data were collected using a sociodemographic questionnaire developed for this study with sociodemographic variables of gender, age, educational level, and employment status at the time of the interview. Semi-structured interviews were conducted using a script with questions regarding caregivers’ experiences in relation to providing care and support for a person with a major lower limb amputation due to vascular disease after hospital discharge and returning home (Chart 1).

**Chart 1:** Question script – Vila Nova de Gaia, VNG, Portugal, 2022–2023.

As a family caregiver of a patient with a lower limb amputation, how do you feel? What changes have occurred in your life since taking on the role of caregiver? Can you tell me how you were prepared to take on this role and provide care? What difficulties have you experienced? Do you still face difficulties now? If so, what are they? When your family member returned home, who helped you overcome the challenges you faced? Do you feel that the preparation provided during the hospital stay was sufficient, or would you have preferred a different training? In caring for your family member, do you feel the need for additional training and support? What do you consider important and what should be improved to help people in your situation overcome the challenges you faced when returning home with a dependent person?

The main researcher scheduled home visits to conduct individual interviews with the family caregiver. After obtaining the signature for the Informed Consent Form, the interviews were digitally audio recorded and later transcribed. Each session lasted approximately 20-25 minutes. Data collection continued until data saturation was achieved.

### Data Analysis and Treatment

Qualitative content analysis was performed using Bardin’s methodology^(14)^ and ATLAS.ti 23.3.4 software (Thomas Muhr, Berlin, Germany). Participants’ anonymity was protected through a coding system consisting of two letters followed by a number. The thematic-categorical approach comprises three main phases.

In the **pre-analysis phase**, researchers conducted a floating reading of the transcriptions to gain an overall understanding and identify preliminary categories, laying the groundwork for further analyses. The **exploration of the material** involved organization of data within ATLAS.ti, where researchers created codes that represented units of meaning and grouped them into thematic categories. The software facilitated the clear visualization of code relationships and insights into theme frequencies, enhancing the comprehension of the content. In the **treatment of the results and interpretation phase**, researchers made inferences based on a theoretical framework.

Each analysis phase was independently conducted by two researchers, with a third consulted in case of discrepancies. Ultimately, through analysis and discussion, researchers reached a consensus on the results. The integration of Bardin’s methodology^(14)^ with ATLAS.ti enables a structured and thorough analysis, leading to meaningful and well-supported findings.

Researcher bias was considered, as personal perceptions could potentially influence the interpretation of data. To minimize this, thematic analysis was conducted by multiple researchers who collaboratively coded the data. This approach ensured triangulation and facilitated collective discussions of various interpretations, ultimately strengthening the validity of the findings.

### Ethical Aspects

The research followed the deontological principles recommended by the Ethics Committee for Studying Human Subjects. The hospital’s ethics committee approved the study and ensured compliance with the Declaration of Helsinki guidelines for self-determination, privacy, anonymity, confidentiality, and informed consent. Participants’ anonymity was preserved by assigning them letters and numbers (FC = Family Caregiver, along with a sequential interview number).

## RESULTS

### Characterization of Participants

The participants in the study were primary caregivers who were family members and had prior experience caring for individuals with dysvascular major lower limb amputation, needing help from family caregivers with daily living activities, and living at home. The study involved 40 participants, with 82.5% of the caregivers being female. The majority of family caregivers were over 60 years old, representing 65% of the group. Caregivers aged between 66 and 70 years made up the largest proportion, accounting for 27.5% of the participants. Considering the educational level, 47,5% had four years of basic education; at the time of the study 37,5% of the participants were active employees. Regarding the relationship between family caregivers and amputees, 65% of them were spouses/partners.

From the content analysis of the interviews, five subcategories emerged: 1) family caregiver role, 2) amputee needs/difficulties, 3) family caregiver needs/difficulties, 4) home transition, and 5) strategies to promote family caregiver empowerment. The categories and subcategories identified in the content analysis of the interviews are summarized in Chart 2.

**Chart 2:** Categories and subcategories emerged from the interviews – Vila Nova de Gaia, VNG, Portugal, 2022–2023.

Categories	Subcategories
**Family caregiver role**	Family caregiver burden Lifestyle changes Accepting amputation Awareness of the family caregiver role Family caregivers’ feelings and attitudes
**Amputee needs/difficulties**	Loss of autonomy and mobility Awareness of health condition Social isolation Need for psychological and mental health support Fear of falling Fitting the prothesis
**Family caregiver needs/difficulties**	Need for psychological and mental health support Family caregivers’ fear of falling Lack of preparation for the role Self-care Need for training/information Need for skills training
**Home transition**	Association and community support Home adaptation Stump care Skills training for family caregivers Family and friends’ support Health professionals support Lack of home support
**Strategies to promote family caregiver empowerment**	Information and training Home support service Technical aids Social and economic support Support/peer group Books and information brochures Home visit Consultation for amputees and caregivers Phone support Longer hospitalization Skills training for family caregivers Psychological and mental health support

### Family Caregiver Role

This category focuses on the experiences and perspectives of family caregivers who are responsible for caring for individuals with dysvascular lower limb amputation. This category includes the following subcategories.

- Family caregiver burden (FC1, FC3, FC4, FC6, FC11, FC15, FC17, FC19, FC21, FC31, FC33, FC35) - “Psychologically, this is exhausting. This was stressful and exhausting. I consider myself a strong person. However, this is very difficult in the first year. However, there are many difficulties. On a psychological level, you get sad. Some days we’re down. We’re tired. Because I have to look after myself at the moment” (FC19).
- Lifestyle changes (FC2, FC4, FC7, FC10, FC14, FC16, FC17, FC18, FC19, FC20, FC23, FC26, FC28, FC31, FC32, FC36, FC38, FC39) - “Changes, in my life was everything. She used to do everything: Now, it’s me. I must do everything, so cleaning the house, cleaning my wife, my personal hygiene, making lunch, dinners, snacks, everything, I do everything.” (FC26).
- Accepting amputation (FC5, FC8, FC16, FC22, FC33, FC34) – “It’s a lot of love for the person themselves. I really really liked it. After seeing that, it was not a lack of a leg. In this case, the patient’s leg was amputated. It is not the lack of a leg because I look at him, as I have always looked at him. I never felt that he had missed a leg’ (FC22).
- Awareness of the family caregiver role (FC9, FC14, FC18, FC33, FC37, FC40) – “I feel an obligation to look after him, to help him, that’s what I do. I would help him, and he would help me a lot, too. But now he needs me to take off his prosthesis, to put it on, to wash it, and to help him bathe’ (FC37).
- Family caregivers’ feelings and attitudes (FC2, FC9, FC12, FC13, FC18, FC22, FC24, FC26, FC27, FC28, FC29, FC33, FC34, FC36, FC38, FC39) – “So the feeling is love? pain? pleasure. I do not know; it is a mixture that we must make, but not out of obligation, out of our will. I have mixed feelings about this situation. It’s a lot” (FC28).

### Amputee Needs/Difficulties

The second category emerged from family caregivers’ perspectives and experiences concerning the care of a person with dysvascular major lower limb amputation and the needs and difficulties that they face after amputation and when returning home. The following subcategories are unfolded in this category:

- Loss of autonomy and mobility (FC3, FC29) – “He was very independent, very active, helped people, was responsible for the cemetery, and decorated my mother’s grave. He would not stop, he always had the gumption, always, always, and he would go to the tides, to the octopuses, to the razor clams, he would always walk, he would not stop; that is, he was unlucky. He stopped being independent and doing things” (FC29).
- Awareness of health condition – (FC22) – “Then, there was the problem that the amputee forgets that he is an amputee, right? He stands up, and there are situations like that. He fell when he left the hospital, when he opened the stump, and even later, he had to go to the hospital to repair some of the stitches.”
- Social isolation (FC21, FC22, FC29, FC38) – “My husband is isolated at home, because the house does not have an elevator, and he only goes out with the firefighters to go to appointments” (FC38).
- Need for psychological and mental support (FC11, FC19, FC20, FC33, FC39) - “If it is at a psychiatric level, for the patient himself. I think about the principles to try, or I do not know, a psychiatrist, or something, because in the case of phantom pain. I know that one night or two, he once told me that he dreamed he had the foot” (FC20).
- Fear of falling (FC34) - “He’s afraid of falling, because when he feels better, he goes to the field, but he’s already fallen twice, so he’s afraid, because the doctor had told him, you have to be very careful, because if you fall, I don’t know how it’s going to be afterwards” (FC34).
- Fitting the prosthesis (FC19) - “He was not seen by the rehabilitation doctor to find out if he was okay with the prosthesis if he fitted it well, if he had to make changes, nothing. He only prescribed a prosthesis that was not compatible with him, and the national health service spent quite a lot, I do not know how many thousand euros, because that is expensive, that is over three thousand euros for sure. The national health system spent this money for this to left aside there.”

### Family Caregiver Needs/Difficulties

This category describes the needs and difficulties that family caregivers experience when caring for a person with dysvascular major lower amputation living at home. This category includes the following subcategories.

- Need for psychological and mental health support (FC18, FC29, FC39, FC40) – “It was very bad. I can say that this was one of the worst phases of my life. We had never been so shocked. I lacked support and felt very lonely. I went to see a psychologist to provide me support in this situation that was happening to me’ (FC40).
- Family caregivers’ fear of falling (FC17, FC23, FC31, FC33, FC35, FC38) – “I was just afraid that if I lifted him up, he would fall on me. He sometimes fell and opened some of the stitches and so on. It was my fear. My only difficulty was the fear that he would fall on me” (FC17).
- Lack of preparation for the role (FC5, FC6, FC9, FC13, FC18, FC34, FC39, FC40) – “I think it was a little bit of miscommunication, someone could call us and tell us something like: look, you are going to do this, because you have to buy this, or you have to buy that, or social security helps with this” (FC9).
- Self-care (FC3, FC5, FC6, FC12, FC10, FC14, FC24, FC25, FC26, FC27, FC28, FC30, FC31, FC32, FC34, FC35, FC38) – “I had some difficulty bathing him because he was an amputee. The lack of balance and everything was a problem, later we bought a proper seat to put him in the shower tray” (FC34).
- Need for training/information (FC1, FC5, FC10, FC11, FC16, FC17, FC18, FC28, FC36, FC39) – “I feel that there is a need for more information, to inform people, to tell them you are entitled to do this, you are entitled to this support and you can request this support more. More knowledge or more training” (FC1).
- Need for skills training (FC29) – “I would like the nurse to teach me how to do it because he will always be an amputee. Even in terms of bathing, even if it is a bedridden person. There are things, there are little tricks that we don’t know because at the time when I took him, he came in a diaper, the person turned to one side, put on the diaper and turned to the other, things like that, as we saw.”

### Home Transition

The present category describes family caregivers’ experiences in providing care during the transition to home after the discharge of a person with dysvascular major lower limb amputation. The following subcategories emerged in this category:

- Association and community support (FC35) – “That’s how it is, the people from the day center come to help bathe him, from Sunday to Sunday. I am the one who pays with my money, and I want it from Sunday to Sunday because hygiene is very important. I could not bathe her by myself either”.
- Home adaptation (FC1, FC12, FC14, FC16, FC19, FC21, FC27, FC35, FC38) - “It was adapting the room, adapting the bathroom, and giving him the best, because we have spent a lot of money, and we are just two retirees…”(FC27).
- Stump care (FC11) – “But before having the prosthesis, it had to be bandaged, with a bandage, in which you had to bandage the whole stump, because it was straighter, so that it wouldn’t open so much or became too flat.”
- Skills training for family caregivers (FC31) – “I went to the hospital to learn a little bit about how to deal with him, in bed, and nothing else. I went a few times. I went a few times. I learned to turn him on the bed and put the diaper, but of course, he must have strength, but he has changed.
- Family and friends support (FC1, FC2, FC4, FC6, FC8, FC9, FC11, FC12, FC13, FC16, FC18, FC19, FC20, FC23, FC24, FC25, FC28, FC30, FC33, FC34, FC35, FC36, FC39, FC40) – “Me and my daughter, and the rest was me. It was only the family nucleus that helped me; I did not have any more help. My daughter and I looked at each other, and also, my son-in-law helped us” (FC28).
- Health professionals’ support (FC13) – “I knew I could count on my physiotherapist, who was always very supportive. She told me about the transfers, how she was really doing, look, and you do it like this, and like this. She basically reinforced what I was beginning to see. “
- Lack of home support (FC34, FC39) – “No one ever asked me, for example, in the hospital if I needed follow-up; if I needed to talk to someone, no one ever went to me; I turned because I felt that need, and that is, I went at my own expense” (FC39).

### Strategies to Promote Family Caregiver Empowerment

This category emerged from the experiences and perspectives of family caregivers regarding strategies to promote empowerment regarding the care of a person with dysvascular major lower limb amputation. The subcategories are as follows.

- Information and training (FC1, FC3, FC8, FC9, FC24, FC26, FC27, FC29) - (…) There could have been more detailed information, I think, there could have been.” Some information from a nurse or doctor could be given, possibly, or they could ask if I was able to do this or that, and that should have been done “(FC8).
- Home support service (FC13, FC31, FC32, FC33, FC34, FC40) – “If someone came to the house and told me, do it this way or do that, that would always be good. I could even be doing something and learn to do it more easily if I had someone to explain it to me. It was necessary for someone to show up at the house” (FC32).
- Technical aids (FC1, FC39) – “For example, if you do not have an articulated bed and you cannot afford it, someone should dispense with one, you do not have a wheelchair and you need it, someone should get one’ (FC1).
- Social and economic support (FC15, FC26) - “In other words, if there is no social service, at least there should be a financial benefit, in which people can pay someone to help take care of the amputee” (FC15).
- Support/peer group (FC39) – “I was also part of a group of people’s family members that was also a university group (...) Okay because sometimes this exchange between people who are sharing the same difficulties the challenges, I think it helps us to fit in and easily solve some problems.”
- Books and information brochures (FC1, FC3, FC13) – “A few small pamphlets, small booklets to bring home with an image, with everything very appealing, that always works very well.” (FC13).
- Home visit (FC1, FC4, FC5, FC6, FC11, FC12, FC14, FC16, FC17, FC19, FC20, FC21, FC23, FC31, FC34, FC35, FC39, FC40) – “We should have that support from a person who came to the house to see how we are doing and If I felt better. Someone who would go and see if I was sick, if I was doing well or badly and call my attention” (FC6).
- Consultation for amputees and caregivers (FC3, FC22, FC27, FC28, FC29, FC32, FC35) – “We should have an appointment at the hospital, to make us more aware of things and to remind us more of things we should do, because there are people who have no idea of the work that this gives, only those who go through this” (FC35).
- Phone support (FC3, FC4, FC5, FC6, FC8, FC14, FC16, FC17, FC18, FC21, FC31, FC33) – “If there was someone who called, at least I could talk a little. A person needs to talk about our sadness and needs to blurt out, but that is it. I don’t talk much. However, everything was missing. It is not worth leaving home. If they called me to ask if I am okay, if I am not doing well, I’d say it, and that is it. A phone call would be enough” (FC31).
- Longer hospitalization (FC33, FC36) – “I would have liked to have had another type of preparation. She should have been hospitalized for a longer time. So, I could prepare myself and learn how to do things.” (FC36).
- Skills training for family caregivers (FC1, FC3, FC19, FC25, FC28, FC33, FC36, FC39) – “I think the important thing is the training, the doing. Seeing people doing. It was important for me that someone would come to the house to help us by training us. It’s important for people who, like me for example, wouldn’t be able to know it...” (FC28).
- Psychological and mental support (FC1, FC3, FC9, FC13, FC18, FC33, FC36) – “On a psychological level, it changes a lot, doesn’t it? With the patient, even with their families. That’s why it’s essential to have support” (FC3).

## DISCUSSION

This study explores the sociodemographic profile of family caregivers of individuals with dysvascular major lower limb amputation, focusing on those in home care settings. Consistent with prior research, the majority of caregivers were women (82.5%), who typically devoted more hours to caregiving than did men. Women also report greater emotional and physical burdens, along with poorer psychological health, highlighting the gender disparity in caregiving that reinforces social inequalities and limits professional opportunities^(15,16)^. The study also found that 65% of caregivers were over 60 years old, with the largest group (27.5%) aged between 66 and 70 years. Older caregivers face heightened health and financial challenges, including an increased risk of chronic illness and limited self-care time^(17,18)^. Nearly half (47.5%) had low educational levels, and those with inadequate health literacy (HL) were more common among spousal caregivers than among offspring. Female gender, older age, and lower education level were independent predictors for low HL, which may be linked to worse outcomes for care recipients^(19)^. Furthermore, 65% of caregivers were spouses or partners, and spousal caregiving was associated with strained marital relationships and a higher risk of depression, especially during transitions into or out of the caregiver role^(20,21)^.

The results of this study highlight the experiences and perspectives of family caregivers regarding their transition to the role of family caregiver for a person with dysvascular major lower limb amputation. According to Meleis’s Transitions Theory, family caregivers undergo significant changes as they take on new roles, requiring the development of new skills and behaviors, often accompanied by emotional challenges such as loss, isolation, and anxiety. These emotional responses are influenced by social support and hindered by barriers such as stigma and emotional overload^(11,22,23)^. Our study supports this theory, with caregivers reporting substantial caregiver burden, including fatigue, psychological exhaustion, and difficulty accepting amputation, all of which contribute to their emotional strain. Most caregivers in our study were women over 60 years old and spouses—groups that are particularly vulnerable to caregiver burden. Factors such as age, gender, and socioeconomic status are widely acknowledged to significantly impact mental health and coping strategies^(24)^. Female spousal caregivers, in particular, often manage multiple roles, and they also avoid seeking for help, and experience higher levels of burden and depressive symptoms^(23)^. Caregivers also reported growing awareness of their role and the emotional toll it entails, expressing mixed feelings of duty, overwhelming, and isolation. These findings highlight the need for effective coping strategies to reduce caregiver burden and enhance physical and psychological well-being^(25,26)^.

During the transition to the family caregiver role, our study participants highlighted their awareness of becoming a caregiver for someone with dysvascular major lower limb amputation. Understanding the resilience of family members and their engagement in self-care is essential as these factors can help mitigate caregiver burden. Educating caregivers on the importance of self-care is crucial, as a stronger focus on self-care and resilience has been associated with reduced caregiver burden^(27)^. Nurses can support caregivers by evaluating their transition status, offering education, fostering social support, and validating their role, turning the experience into an opportunity for growth and resilience while encouraging positive adaptation and confidence in their new identity^(11)^.

The participants in the present study highlighted the needs and difficulties that amputees face and the impact of care; they reported a lack of awareness of health condition as a problem. Awareness is a key aspect of transition, evident in how well an individual’s knowledge of the process aligns with their responses and perceptions during the transition^(23)^.

Loss of autonomy and mobility were identified in this study by family caregivers as a difficulty faced by dysvascular major lower limb amputees. The primary functional challenge for lower limb amputees is their limited mobility in daily life. The increased dependence is a consequence of this restriction^(1)^.

Family caregivers also reported that amputees face difficulties related to fitting prothesis and social isolation. The mobility constraints experienced by amputees can be primarily attributed to the use of prostheses that do not fit properly, and a diminished level of trust in their prosthesis. This restriction can lead to a higher level of dependence, ultimately reducing accessibility to the workplace, education, social interactions, and daily life^(1)^.

Participants reported as well that another difficulty amputees face is the fear of falling. Falls are linked to the fear of falling and reduced balance confidence in people with limb loss. This decreased balance confidence is associated with lower levels of prosthetic function^(28)^. Family caregivers also additionally highlighted a need for psychological and mental health support for amputees. The mental health of amputees should be managed throughout their entire recovery period, even before amputation. However, in practice, they often do not receive outpatient mental health support until they report psychological issues^(29)^.

A main category that emerged from our study was family caregiver needs/difficulties, with subcategories related to need for psychological and mental health support, need for training/information and need for skills training. Caregivers need skills training and psychological support, and institutions should develop services to enhance caregiver support, improve educational outcomes, and elevate the quality of life of both caregivers and patients, especially those with substantial psychological needs^(30)^. Family caregivers reported having difficulties with fear of falling. Concerns regarding falls among caregivers can have a detrimental impact on their physical and mental well-being, exacerbate the burden of caregiving, and hinder efforts to prevent falls at home^(31)^. The study findings demonstrated that family caregivers experience difficulties in supporting self-care activities, with a lack of preparation for the role identified as a significant challenge. The informal caregiver training process focuses on assessing the caregiver’s ability to provide care for someone with self-care dependence, which helps identify specific training needs^(32)^.

Another main category identified in our study was home transition. Participants described their experiences of returning home after hospital discharge and subcategories emerged related to the social support available to family caregivers returning to home/community: these included association and community support, home adaptation, family and friends support, health professional support, and lack of home support. Having a support network is crucial for family caregivers as it helps alleviate the burden and stress of caregiving^(33)^. Caregivers can find relief and support through community resources, enabling them to meet the diverse needs of patient care^(34)^.

When transitioning home, family caregivers referred that they had skills training regarding activities of caring for a dysvascular major lower limb amputee and at home assumed the stump care to prepare for prothesis. Caregiver assessments are essential to determine their ability to assume roles and identify the necessary training required. Providing education and skill training can enhance caregivers’ confidence and improve their ability to handle daily care challenges effectively^(35)^. Transitioning from hospital to home after major lower limb amputation due to vascular disease can be emotionally challenging for patients and families. Feeling informed, involved, and supported is essential. Integrated care programs, such as the Danish Safe Journey, provide reassurance and a sense of safety for amputees and their families^(36)^.

The last category that emerged from our findings concerned strategies to promote family caregiver empowerment concerning strategies that cannot enhance empowerment when returning home. These strategies appear in the following subcategories: information and training, home support service, technical aids, social and economic support, support/peer groups, books and information, home visit, consultation for amputees and caregivers, longer hospitalization, skills training for family caregivers, and psychological and mental health support. This type of strategy can be used in the construction of interventions and programs to promote the empowerment of family caregivers of dysvascular major lower limb amputees’ transition to home care.

Health literacy and self-efficacy of caregivers can be improved by interventions based on a family centered empowerment approach, assisting caregivers in providing specialized and efficient care and leading to improved quality of care in caregiving^(37)^. Caregivers benefit from enhanced knowledge of the disease, their responsibilities, and available resources. After addressing their informational needs, they can gain further from problem-solving training and interventions aimed at managing care recipient behaviors and their own emotional responses. Effective interventions should address both practical and emotional aspects of caregiving^(9)^.

Nurse-led home-based interventions can significantly improve the health-related quality of life of caregivers of patients with chronic or disabling conditions in the community^(38)^. Intervention programs can help reduce caregiver burden across different health conditions. Psychoeducational interventions for caregivers of hemodialysis patients have shown positive effects on both the burden and quality of life^(39)^. Likewise, educational, supportive, and psychological interventions have proven effective in alleviating the burden among caregivers of chronic kidney disease patients^(40)^.

This study had some limitations that should be considered when interpreting the findings. First, it focuses solely on family caregivers, which may not fully capture the perspectives of dysvascular major lower limb amputees or healthcare providers involved in the caregiving process. The research was conducted at a single vascular surgery unit in northern Portugal, which may limit the generalizability of the results to other regions or healthcare settings. While the broader doctoral research project includes inputs from nurses and amputees, the specific geographic and institutional context of the sample may not reflect national or international trends. Additionally, the use of semi-structured interviews, although allowing for in-depth exploration, may have introduced interviewer bias. Despite efforts to mitigate bias, researchers’ perspectives can still influence data interpretation, even with multiple coders. Finally, the study’s cross-sectional design limits its ability to draw causal conclusions.

Future research should include multisite studies with longitudinal designs to capture a more comprehensive range of caregiving experiences. Longitudinal studies are also crucial to examine how outcomes evolve over time, providing insights into the long-term impact of care. Expanding the participant pool to include family caregivers from diverse socioeconomic backgrounds and varying demographic characteristics will further strengthen the evidence and broaden the relevance of our findings.

This study emphasizes the need for family centered empowerment programs and interventions to support family caregivers of dysvascular major lower limb amputees. To reduce the caregiver burden and promote empowerment, clear pathways for accessing training, community support, and psychological services are essential. Hospitals should establish referral systems for caregiver training, offer workshops (online or in-person), and provide necessary resources such as technical aids.

Coordination between hospitals and primary healthcare is crucial to ensure continuity of care, connecting caregivers to community resources, such as support groups, social and financial aid, and home care services, with ongoing follow-up and accessible support. Psychological services, including phone counseling for rural caregivers, should also be easily accessible. These initiatives can guide policy development, optimize resource allocation, and ensure that caregivers receive adequate support during hospital-to-home transition. Nurses play a key role in educating caregivers on daily care, self-care, stump management, coordinating care, and referring them to community resources, thus ensuring that caregivers feel empowered and supported.

To enhance the practical value of these findings, it is crucial to detail their implementation and evaluation. Healthcare providers and policymakers can integrate these interventions into care models by standardizing referral processes, incorporating caregiver education into discharge planning, and securing community resources through formal partnerships. Regular caregiver feedback can be used to evaluate the effectiveness of training, psychological support, and peer groups, enabling ongoing improvements. Clear steps for integration and evaluation will strengthen the real-world applications of family caregiver empowerment initiatives.

## CONCLUSION

Family caregivers play a crucial role in supporting amputees, providing both emotional and physical care while managing significant lifestyle changes and emotional challenges. They often face isolation, fear, and mental health struggles, underscoring the need to prioritize their well-being. Empowering caregivers requires a comprehensive approach, including educational resources, skills training, home support services, and access to social and psychological support. Strengthening social networks and addressing emotional well-being are essential to improve the quality of life for both caregivers and amputees.

This research highlights the profound impact of dysvascular major lower limb amputation on caregivers and the critical need for comprehensive support systems to address their evolving needs during the transition from hospital to their home. The findings emphasize the importance of developing family-centered empowerment programs and interventions that integrate hospital-based strategies, such as caregiver training and skills development for daily care tasks and self-care, with community-based interventions, including financial support, peer networks, and mental health care.

Future research should explore the implementation and effectiveness of these programs to assess their potential for reducing caregivers’ burden, fostering empowerment, and improving outcomes for both caregivers and amputees.

## Data Availability

Data from this study are available upon request from the corresponding author.
