# Supplementary material for: Experiences and challenges of family caregivers of people with lower limb amputation: a qualitative study
Source: Rev Esc Enferm USP. 2025 May 26;59:e20240264. doi: 10.1590/1980-220X-REEUSP-2024-0264en (PMC12118359; doi:10.1590/1980-220X-REEUSP-2024-0264en)
Supplement: Translation

# Translation: Experiências e desafios de cuidadores familiares de pessoas com amputação de membros inferiores: estudo qualitativo

**DOI:** 10.1590/1980-220X-REEUSP-2024-0264pt

**Authors:** Diana Fonseca Rodrigues, António Luís Rodrigues Faria de Carvalho, Cristina Maria Correia Barroso Pinto

**Affiliations:** 1 Universidade do Porto, Faculdade de Medicina, RISE-Health, Porto, Portugal.; 2 Universidade do Porto, Instituto de Ciências Biomédicas Abel Salazar, Porto, Portugal.; 3 Escola Superior de Enfermagem do Porto, Porto, Portugal.

**Keywords:** Amputados, Empoderamento, Cuidadores, Transição do Hospital para o Domicílio, Extremidade Inferior, Pesquisa Qualitativa

## Abstract

**Objetivo:**

Explorar as experiências de cuidadores familiares em transição para seus papéis de cuidadores de pessoas com amputação maior de membros inferiores de causa vascular, identificar os desafios que enfrentam, entender suas necessidades específicas durante a transição do hospital para a domicílio, examinar suas experiências durante essa transição e reunir *insights* sobre estratégias que promovem seu empoderamento no cuidado.

**Método:**

Estudo exploratório transversal, descritivo, com abordagem qualitativa, em que foram realizadas entrevistas semiestruturadas com 40 cuidadores familiares de indivíduos com amputação disvascular maior de membros inferiores. Os dados foram analisados por meio do *software* ATLAS.ti e do método de análise de conteúdo de Bardin.

**Resultados:**

A análise de conteúdo identificou cinco categorias: 1) papel do cuidador familiar, 2) necessidades/dificuldades do amputado, 3) necessidades/dificuldades do cuidador familiar, 4) transição domiciliar e 5) estratégias para promover o empoderamento do cuidador familiar, destacando aspectos críticos da experiência de cuidar e identificando áreas de intervenção.

**Conclusão:**

Os resultados enfatizam a necessidade de programas de capacitação centrados na família, combinando treinamento hospitalar e apoio comunitário. Pesquisas futuras devem avaliar seu impacto na redução da sobrecarga do cuidador e na melhoria dos resultados para cuidadores e amputados.

## INTRODUÇÃO

A amputação de membros representa um desafio significativo para a saúde global, afetando profundamente a vida dos indivíduos e suas famílias. As condições de origem vascular, causadas principalmente por doença arterial periférica e diabetes mellitus, são as principais causas de amputações de membros inferiores^(1,2)^ Mais de 80% das amputações de membros inferiores são devidas a causas vasculares, principalmente por doença arterial periférica e diabetes mellitus ou uma combinação de ambas^(3)^. Um estudo que avaliou a capacidade funcional 12 meses após a amputação de membros inferiores de causa vascular relatou função física ruim, com apenas 39% dos pacientes retornando ao nível prévio de mobilidade^(4)^. A amputação de membros inferiores é um evento que altera a vida e pode impactar negativamente a saúde física e mental dos pacientes^(2)^.

Os resultados de reabilitação após as amputações de causa vascular frequentemente são ruins, com os indivíduos afetados apresentando um nível de incapacidade superior a 95% em relação à população em geral^(5)^. As amputações de membros inferiores podem resultar em limitações físicas que podem restringir as habilidades funcionais dos amputados nas atividades diárias, muitas vezes levando a perda significativa de independência e aumento da dependência de outras pessoas^(1)^. A pessoa dependente é aquela que tem capacidade limitada ou é incapaz de iniciar e realizar atividades essenciais para o bem-estar, saúde e manutenção da vida sem a ajuda de outrem^(6)^. Os indivíduos podem vivenciar situações em que não conseguem atender suficientemente suas necessidades de autocuidado, resultando em déficits de autocuidado. Isso pode ser causado por fatores como doença, lesão, incapacidade ou falta de conhecimento e recursos^(7)^.

Os cuidadores informais desempenham papel crucial no apoio aos familiares, auxiliando nas atividades de vida diária, realizando tarefas médicas e de enfermagem, fornecendo apoio psicossocial e comunicando-se com os profissionais de saúde^(8)^. Cuidadores familiares são parentes, amigos, parceiros ou vizinhos que oferecem apoio não remunerado a indivíduos com limitações físicas, mentais ou cognitivas. A competência, as habilidades e a motivação dos cuidadores podem variar por vários motivos^(9)^. Um estudo anterior mostrou que o cuidado com a amputação cria estresse psicológico, financeiro e físico nas famílias e que a sobrecarga do cuidador aumenta significativamente com a gravidade da amputação, com amputações maiores colocando maior pressão que as menores^(10)^. As tarefas do cuidador aumentam progressivamente de intensidade à medida que a deficiência do destinatário do cuidado piora, tornando-se mais demoradas, complexas e estressantes. Essas demandas crescentes podem levar a respostas emocionais negativas que, por sua vez, podem desencadear reações comportamentais ou físicas, aumentando o risco do cuidador para problemas de saúde mental ou física^(9)^.

Uma mudança na condição de saúde de um membro da família fará com que outros membros assumam o papel de cuidador, além de desencadear uma transição dentro da família. Um familiar que está disposto a assumir a responsabilidade de um cuidador passará por uma transição situacional que trará mudanças tanto em sua própria vida quanto na vida da pessoa dependente. A aprendizagem e a aquisição de novas habilidades são os componentes essenciais para esse processo^(11)^. Apoiar os cuidadores informais é essencial não só para seu bem-estar, mas também para a sociedade. Destacar a carga pesada e as necessidades muitas vezes não atendidas dos cuidadores pode ajudar a comunidade de saúde a se conscientizar sobre o impacto da amputação na família de um paciente^(10)^.

Os cuidadores familiares desempenham papel crítico nos planos de cuidados para pacientes com doenças crônicas. À medida que a dependência do paciente em relação aos cuidadores cresce, os enfermeiros enfrentam desafios para garantir a segurança do paciente e a qualidade do atendimento. Ao fortalecer a comunicação e fornecer suporte robusto, os enfermeiros podem aprimorar a competência dos cuidadores, equipando-os com as habilidades necessárias para manter a segurança do paciente^(12)^. Os enfermeiros podem implementar intervenções para capacitá-los a mitigar a sobrecarga dos cuidadores familiares. Ao contrário dos modelos tradicionais de educação, o empoderamento do cuidador enfatiza o autogerenciamento, transformando a entrega passiva de informações em colaboração ativa entre cuidadores e pacientes^(13)^.

Este estudo faz parte de um projeto de pesquisa de doutorado mais ampla que explora as perspectivas de enfermeiros e amputados maiores de membros inferiores de causa vascular. Tem como objetivo contribuir para o desenvolvimento de intervenções e programas de apoio aos cuidadores familiares na gestão do processo de cuidar e abordar as dimensões física, emocional e social do cuidado. Seus principais objetivos foram:

- Explorar a experiência de transição para o papel de cuidador familiar de uma pessoa com amputação maior de membros inferiores de causa vascular.
- Identificar as dificuldades e os desafios enfrentados por cuidadores familiares no cuidado de uma pessoa com amputação maior de membros inferiores de causa vascular;
- Identificar as necessidades/dificuldades dos cuidadores familiares de uma pessoa com amputação maior de membros inferiores de causa vascular na transição para o papel de cuidador;
- Explorar a experiência de cuidadores familiares de uma pessoa com amputação maior de membros inferiores de causa vascular durante a transição hospital/domicílio.
- Compreender as perspectivas dos cuidadores familiares sobre estratégias para promover seu empoderamento no cuidado à pessoa com amputação maior de membros inferiores de causa vascular.

## MÉTODO

### Desenho e Configurações do Estudo

Estudo exploratório, transversal, descritivo e qualitativo, realizado numa unidade de cirurgia vascular de um hospital do norte de Portugal. Durante o estudo, foram cuidadosamente considerados critérios de pesquisa rigorosos e seguidos o guia COREQ (Critérios Consolidados para Relatar Estudos Qualitativos). Os dados foram coletados entre maio de 2022 e junho de 2023 por um dos pesquisadores com experiência na realização de entrevistas e que não era membro da equipe da unidade.

### Seleção dos Participantes

Os participantes foram selecionados entre cuidadores familiares que acompanhavam pacientes com amputação disvascular maior de membros inferiores em consulta de acompanhamento hemodinâmico de doenças vasculares num Serviço de Cirurgia Vascular de um hospital do Norte de Portugal.

Os cuidadores familiares foram recrutados de acordo com os seguintes critérios: 1) ter 18 anos ou mais; 2) prestar assistência domiciliária a um amputado maior de membro inferior de causa vascular; 3) ser identificado como cuidador primário que cuidava do amputado desde a alta e 4) cuidar de amputados que necessitavam de assistência nas atividades de vida diária.

A coleta de dados foi realizada por 13 meses, visando toda a população de familiares cuidadores de pacientes com amputações maiores de membros inferiores devido à doença arterial periférica (DAP) que compareceram às consultas de acompanhamento hemodinâmico pós-amputação para doenças vasculares. A seleção dos participantes baseou-se na saturação dos dados, que ocorreu após a realização de 40 entrevistas. A saturação é definida como o ponto em que dados adicionais não podem mais contribuir para novos *insights* para o fenômeno em estudo, levando à redundância. Segundo Bardin^(14),^, durante a categorização e a análise os pesquisadores devem estar atentos para reconhecer quando os dados deixam de fornecer novos elementos, marcando a obtenção da saturação, o que foi confirmado neste estudo após 40 entrevistas.

Foram identificados potenciais vieses e tomadas medidas para os mitigar. Uma das principais preocupações foi o viés de seleção, pois os participantes podem ter sido selecionados com base em sua disponibilidade ou vontade de participar. Para minimizar esse viés, foram feitos esforços para incluir indivíduos de diferentes faixas etárias e origens socioeconômicas. Embora o recrutamento não tenha sido realizado em vários contextos, essa abordagem ajudou a garantir uma amostra mais variada e representativa de cuidadores familiares.

### Coleta de Dados

Os dados foram coletados por meio de um questionário sociodemográfico desenvolvido para este estudo com variáveis sociodemográficas de sexo, idade, escolaridade e situação de trabalho no momento da entrevista. Foram realizadas entrevistas semiestruturadas mediante um roteiro com perguntas sobre as experiências dos cuidadores em relação ao cuidado e apoio a uma pessoa com amputação importante de membro inferior devido a doença vascular após a alta hospitalar e o retorno para casa (Quadro 1).

**Quadro 1:** Roteiro de perguntas – Vila Nova de Gaia, VNG, Portugal, 2022–2023.

Como você se sente como cuidador familiar de um paciente com amputação de membro inferior? Que mudanças ocorreram em sua vida desde que assumiu o papel de cuidador? Como você estava preparado para assumir esse papel e prestar cuidados? Que dificuldades você experimentou? Você ainda enfrenta dificuldades agora? Em caso afirmativo, quais são? Quando seu familiar voltou para casa, quem o ajudou a superar os desafios que enfrentou? Você acha que a preparação fornecida durante a internação foi suficiente ou teria preferido um treinamento diferente? Ao cuidar de seu familiar, você sente a necessidade de treinamento e apoio adicionais? O que você considera importante e o que deve ser melhorado para ajudar as pessoas em sua situação a superar os desafios que você enfrentou ao voltar para casa com uma pessoa dependente?

A pesquisadora principal agendou visitas domiciliárias para realização de entrevistas individuais com o cuidador familiar. Após a obtenção da assinatura do Termo de Consentimento Livre e Esclarecido, as entrevistas foram gravadas digitalmente em áudio e posteriormente transcritas. Cada sessão durou aproximadamente 20 a 25 minutos. A coleta de dados continuou até que a saturação dos dados fosse alcançada.

### Análise e Tratamento dos Dados

A análise qualitativa de conteúdo foi realizada por meio da metodologia de Bardin^(14)^, com auxílio do *software* ATLAS.ti 23.3.4 (Thomas Muhr, Berlim, Alemanha). O anonimato dos participantes foi protegido por meio de um sistema de codificação composto por duas letras seguidas de um número.

A abordagem temático-categórica compreendeu três fases principais. Na **fase de pré-análise**, os pesquisadores realizaram uma leitura flutuante das transcrições para obter uma compreensão geral e identificar categorias preliminares, lançando as bases para análises posteriores. A **exploração do material** envolveu a organização dos dados dentro do ATLAS.ti, ocasião em que os pesquisadores criaram códigos para representar unidades de significado e os agruparam em categorias temáticas. O *software* facilitou a visualização clara das relações de código e *insights* sobre as frequências do tema, melhorando a compreensão do conteúdo. Na **fase de tratamento dos resultados e interpretação**, os pesquisadores fizeram inferências com base em um referencial teórico.

Cada fase de análise foi conduzida de forma independente por dois pesquisadores, sendo um terceiro consultado em caso de discrepâncias. Por meio de análise e discussão, os pesquisadores chegaram a um consenso sobre os resultados. A integração da metodologia de Bardin^(14)^ com o ATLAS.ti permitiu uma análise estruturada e minuciosa, levando a achados significativos e bem fundamentados.

O viés do pesquisador foi considerado, pois as percepções pessoais podem influenciar a interpretação dos dados. Para minimizá-lo, a análise temática foi conduzida por vários pesquisadores que codificaram os dados de forma colaborativa. Essa abordagem garantiu a triangulação e facilitou discussões coletivas de várias interpretações, fortalecendo a validade dos achados.

### Aspectos Éticos

A pesquisa seguiu os princípios deontológicos preconizados pelo Comitê de Ética para o Estudo de Seres Humanos. O comitê de ética do hospital aprovou o estudo e garantiu o cumprimento das diretrizes da Declaração de Helsinque para autodeterminação, privacidade, anonimato, confidencialidade e consentimento informado. O anonimato dos participantes foi preservado com a atribuição das letras CF (Cuidador Familiar), juntamente com um número sequencial da entrevista.

## RESULTADOS

### Caracterização dos Participantes

Participaram do estudo cuidadores primários que eram membros da família, residiam no mesmo domicílio que o paciente e tinham experiência prévia no cuidado de indivíduos com amputação maior de membros inferiores de causa vascular que necessitavam de ajuda de cuidadores familiares nas atividades de vida diária. O estudo envolveu 40 cuidadores, 82,5% dos quais era do sexo feminino e 65,0% eram cônjuges/companheiros do paciente. A maioria (65,0%) tinha mais de 60 anos e aqueles com idades entre 66 e 70 anos constituíram a maior proporção, representando 27,5% dos participantes. Quanto ao nível de escolaridade, 47,5% possuíam quatro anos de ensino fundamental. No momento do estudo, 37,5% eram funcionários ativos.

A análise de conteúdo das entrevistas permitiu identificar cinco subcategorias: 1) papel do cuidador familiar, 2) necessidades/dificuldades do amputado, 3) necessidades/dificuldades do cuidador familiar, 4) transição domiciliar e 5) estratégias para promover o empoderamento do cuidador familiar. As categorias e subcategorias identificadas na análise de conteúdo das entrevistas estão resumidas no Quadro 2.

**Quadro 2:** Categorias e subcategorias identificadas – Vila Nova de Gaia, VNG, Portugal, 2022–2023.

Categorias	Subcategorias
**Papel do cuidador familiar**	Sobrecarga do cuidador familiar Mudanças no estilo de vida Aceitando a amputação Consciência do papel de cuidador familiar Sentimentos e atitudes dos cuidadores familiares
**Necessidades/dificuldades do amputado**	Perda de autonomia e mobilidade Consciência da condição de saúde Isolamento social Necessidade de apoio psicológico e de saúde mental Medo de cair Adaptação da prótese
**Necessidades/dificuldades do cuidador familiar**	Necessidade de apoio psicológico e de saúde mental Medo de cair dos cuidadores familiares Falta de preparo para o papel Autocuidado Necessidade de formação/informação Necessidade de treinamento de habilidades
**Transição domiciliar**	Apoio da associação e da comunidade Adaptação ao domicílio Cuidados com o coto. Treinamento de habilidades para cuidadores familiares Apoio da família e amigos Apoio dos profissionais de saúde Falta de apoio domiciliar
**Estratégias para promover o empoderamento do cuidador familiar**	Informação e treinamento Serviço de apoio domiciliário Ajudas técnicas Apoio social e econômico Grupo de apoio/pares Livros e folhetos informativos Visita domiciliária Consulta para amputados e cuidadores Suporte por telefone Maior tempo de internação Treinamento de habilidades para cuidadores familiares Apoio psicológico e de saúde mental

### Papel de Cuidador Familiar

Esta categoria enfoca as experiências e as perspectivas dos cuidadores familiares responsáveis pelo cuidado de indivíduos com amputação de membros inferiores de causa vascular e inclui as seguintes subcategorias.

- Sobrecarga do cuidador familiar (CF1, CF3, CF4, CF6, CF11, CF15, CF17, CF19, CF21, CF31, CF33, CF35) - “Psicologicamente, isso é desgastante. Isso foi estressante e exaustivo. Eu me considero uma pessoa forte. No entanto, isso é muito difícil no primeiro ano. No entanto, existem muitas dificuldades. Em um nível psicológico, você fica triste. Alguns dias estamos para baixo. Estamos cansados. Porque eu tenho que me cuidar no momento” (CF19).
- Mudanças no estilo de vida (FC2, FC4, FC7, FC10, FC14, FC16, FC17, FC18, FC19, FC20, FC23, FC26, FC28, FC31, FC32, FC36, FC38, FC39) - “Mudanças, na minha vida ela era tudo. Ela costumava fazer tudo: agora, sou eu. Devo fazer tudo, então limpar a casa, limpar minha esposa, minha higiene pessoal, fazer almoço, jantar, lanches, tudo, eu faço tudo.” (CF26).
- Aceitando a amputação (FC5, FC8, CF16, FC22, FC33, FC34) – “É muito amor pela própria pessoa. Eu realmente gostei. Depois de ver isso, não foi falta de perna. Nesse caso, a perna do paciente foi amputada. Não é a falta de uma perna porque eu olho para ele como sempre olhei .-Eu nunca senti que ele tinha perdido uma perna’ (CF22).
- Consciência do papel de cuidador familiar (CF9, CF14, CF18, CF33, CF37, CF40) – “Eu sinto a obrigação de cuidar dele, ajudá-lo, é o que eu faço. Eu o ajudaria e ele me ajudaria muito também. Mas agora ele precisa que eu tire a prótese, coloque, lave e ajude a tomar banho’ (CF37).
- Sentimentos e atitudes dos cuidadores familiares (CF2, CF9, CF12, CF13, CF18, CF22, CF24, CF26, CF27, CF28, CF29, CF33, CF34, CF36, CF38, CF39) – “Então o sentimento é amor? Dor? Prazer? Eu não sei. É uma mistura do que devemos fazer, mas não por obrigação, por nossa vontade. Tenho sentimentos mistos sobre esta situação. É muito” (CF28).

### Necessidades/Dificuldades do Amputado

A segunda categoria emergiu de perspectivas e experiências dos cuidadores familiares sobre o cuidado à pessoa com amputação maior de membros inferiores de causa vascular e as necessidades e as dificuldades que enfrentaram após a amputação, no retorno para a casa. As seguintes subcategorias são desdobradas nesta categoria:

- Perda de autonomia e mobilidade (CF3, CF29) – “Ele era muito independente, muito ativo, ajudava as pessoas, era responsável pelo cemitério e decorava o túmulo da minha mãe. Ele não parava, sempre tinha bom senso, sempre, sempre, e ia para as marés, para os polvos, para os lingueirão, sempre andava, não parava; isto é, ele não teve sorte. Ele deixou de ser independente e de fazer coisas” (CF29).
- Consciência da condição de saúde (CF22) – “Aí, teve o problema de o amputado esquecer que é amputado, né? Ele se levanta e há situações como essa. Ele caiu quando saiu do hospital, quando abriu o coto e, mesmo depois, teve que ir ao hospital para consertar alguns dos pontos.
- Isolamento social (CF21, CF22, CF29, CF38) – “Meu marido fica isolado em casa, porque a casa não tem elevador e ele só sai com os bombeiros para ir às consultas” (CF38).
- Necessidade de apoio psicológico e mental (CF11, CF19, CF20, CF33, CF39) - “Se for a nível psiquiátrico, para o próprio doente. Eu penso sobre os princípios para tentar, não sei, um psiquiatra ou algo assim, porque no caso da dor fantasma... Eu sei que em uma noite ou duas ele me disse uma vez que sonhou que estava com o pé” (CF20).
- Medo de cair (CF34) - “Ele tem medo de cair, porque quando se sente melhor, ele vai para o campo, mas já caiu duas vezes. Então ele tem medo, porque o médico tinha falado para ele: Você tem que ter muito cuidado, porque se cair, eu não sei como vai ser depois” (CF34).
- Colocação da prótese (CF19) - “Ele não foi atendido pelo médico de reabilitação para saber se estava bem com a prótese, se ele a colocou bem, se ele teve que fazer trocas, nada. Só receitou uma prótese que não era compatível com ele e o serviço nacional de saúde gastou bastante, não sei quantos mil euros, porque isso é caro, são mais de três mil euros com certeza. O sistema nacional de saúde gastou esse dinheiro para que isso fosse deixado de lado lá.

### Necessidades/Dificuldades do Cuidador Familiar

Esta categoria descreve as necessidades e as dificuldades que os cuidadores familiares vivenciam ao cuidar de uma pessoa com amputação maior menor de causa vascular e que mora no mesmo domicílio. Inclui as seguintes subcategorias.

- Necessidade de apoio psicológico e de saúde mental (CF18, CF29, CF39, CF40) – “Foi muito ruim. Posso dizer que esta foi uma das piores fases da minha vida. Nunca ficamos tão chocados. Eu não tinha apoio e me sentia muito sozinha. Fui procurar uma psicóloga para me dar apoio nessa situação que estava acontecendo comigo’ (CF40).
- Medo de cair dos cuidadores familiares (CF17, CF23, CF31, CF33, CF35, CF38) – “Eu só tinha medo de que, se eu o levantasse, ele caísse em cima de mim. Ele às vezes caía e abria alguns dos pontos e assim por diante. Era o meu medo. Minha única dificuldade era o medo de que ele caísse em cima de mim” (CF17).
- Falta de preparo para o papel (CF5, CF6, CF9, CF13, CF18, CF34, CF39, CF40) – “Eu acho que foi um pouco de falta de comunicação. Alguém poderia ligar para a gente e falar algo assim: Olha, você vai fazer isso, porque você tem que comprar isso ou você tem que comprar aquilo, ou a previdência ajuda nisso” (CF9).
- Autocuidado (CF3, CF5, CF6, CF12, CF10, CF14, CF24, CF25, CF26, CF27, CF28, FC30, FC31, CF32, CF34, CF35, CF38) – “Tive alguma dificuldade de dar banho nele porque ele era amputado. A falta de equilíbrio e tudo foi um problema, depois compramos um assento próprio para colocá-lo na base do chuveiro” (CF34).
- Necessidade de formação/informação (FC1, FC5, FC10, FC11, FC16, FC17, FC18, FC28, FC36, FC39) – “Sinto que há necessidade de mais informação, de informar as pessoas, de lhes dizer que têm direito a fazer isto, têm direito a esse apoio e podem pedir mais apoio. Mais conhecimento ou mais formação” (CF1).
- Necessidade de treinamento de habilidades (CF29) – “Eu gostaria que o enfermeiro me ensinasse como fazer, porque ele sempre será um amputado. Mesmo em termos de banho, mesmo que seja uma pessoa acamada. Tem coisas, tem pequenos truques que a gente não sabe, porque na hora que eu peguei, ele veio de fralda, a pessoa virou-o para um lado, colocou a fralda e virou-o para o outro. Coisas assim, como a gente viu.”

### Transição Domiciliar

A presente categoria descreve as experiências dos cuidadores familiares na prestação de cuidados durante a transição para o domicílio após a alta de uma pessoa com amputação maior de membros inferiores de causa vascular. Nela emergiram as seguintes subcategorias:

- Apoio da associação e da comunidade (CF35) – “É assim, o pessoal do centro-dia vem ajudar a dar banho nele, de domingo a domingo. Sou eu quem paga com meu dinheiro e quero de domingo a domingo porque a higiene é muito importante. Eu também não poderia banhá-lo sozinha”.
- Adaptação domiciliária (CF1, CF12, CF14, CF16, CF19, CF21, CF27, CF35, CF38) - “Foi adaptar o quarto, adaptar o banheiro e dar a ele o melhor, porque a gente gastou muito dinheiro, e somos só dois aposentados...” (CF27).
- Cuidados com o coto (CF11) – “Mas antes de ter a prótese, ela tinha que ser enfaixada, com um curativo, em que você tinha que enfaixar todo o coto, porque era mais reto, para que não abrisse tanto ou ficasse muito plano.”
- Treinamento de habilidades para cuidadores familiares (CF31) – “Fui ao hospital para aprender um pouco sobre como lidar com ele, na cama e nada mais. Eu fui algumas vezes. Eu fui algumas vezes. Aprendi a virá-lo na cama e colocar a fralda, mas é claro que ele deve ter força, mas mudou.
- Apoio da família e dos amigos (FC1, FC2, FC4, FC6, FC8, FC9, FC11, FC12, FC13, FC16, FC18, FC19, FC20, FC23, FC24, FC25, FC28, FC30, FC33, FC34, FC35, FC36, FC39, FC40) – “Eu e minha filha, e o resto era eu. Foi apenas o núcleo familiar que me ajudou. Eu não tive mais ajuda. Eu e minha filha nos olhamos e meu genro também nos ajudou” (CF28).
- Apoio dos profissionais de saúde (CF13) – “Eu sabia que podia contar com minha fisioterapeuta, que sempre me apoiou muito. Ela me contou sobre as transferências, como ela estava realmente se saindo: Olha, você faz assim e assim. Ela basicamente reforçou o que eu estava começando a ver”.
- Falta de apoio no domicílio (CF34, CF39) – “Ninguém nunca me perguntou no hospital, por exemplo, se eu precisava de acompanhamento. Se eu precisasse falar com alguém, ninguém nunca ia até mim. Eu me virei porque senti essa necessidade, ou seja, fui às minhas próprias custas” (CF39).

### Estratégias Para Promover o Empoderamento do Cuidador Familiar

Esta categoria emergiu das vivências e perspectivas dos cuidadores familiares sobre estratégias para promover o empoderamento no cuidado de uma pessoa com amputação maior de membros inferiores de causa vascular. As subcategorias são as seguintes.

- Informação e formação (FC1, FC3, FC8, FC9, FC24, FC26, FC27, FC29) – “Poderia ter havido informações mais detalhadas, eu acho, poderia ter havido. Alguma informação de uma enfermeira ou médico poderia ser dada, possivelmente, ou eles poderiam perguntar se eu conseguia fazer isso ou aquilo, e aquilo que deveria ter sido feito.” (CF8).
- Serviço de apoio domiciliário (FC13, FC31, FC32, FC33, FC34, FC40) – “Se alguém viesse a casa e me dissesse, faz assim ou aquilo, isso seria sempre bom. Eu poderia até estar fazendo algo e aprender a fazê-lo mais facilmente se tivesse alguém para me explicar. Era necessário que alguém aparecesse na casa” (CF32).
- Ajudas técnicas (CF1, CF39) – “Por exemplo, se você não tem cama articulada e não tem condições de pagar, alguém deve dispensar uma, se você não tem cadeira de rodas e você precisa, alguém deve pegar” (CF1).
- Apoio social e econômico (CF15, CF26) - “Ou seja, se não tem serviço social, pelo menos deveria ter um benefício financeiro em que as pessoas possam pagar alguém para ajudar a cuidar do amputado” (CF15).
- Grupo de apoio/pares (FC39) – “Eu fazia parte de um grupo de familiares de pessoas que também era um grupo universitário. OK, porque às vezes essa troca entre pessoas que estão compartilhando as mesmas dificuldades, os desafios, acho que nos ajuda a nos encaixar e resolver facilmente alguns problemas.”
- Livros e brochuras informativas (FC1, FC3, FC13) – “Alguns pequenos folhetos pequenos livretos para levar para casa com uma imagem, com tudo muito apelativo, que funciona sempre muito bem.” (CF13).
- Visita domiciliária (FC1, FC4, FC5, FC6, FC11, FC12, FC14, FC16, FC17, FC19, FC20, FC21, FC23, FC31, FC34, FC35, FC39, FC40) – “Devemos ter esse apoio de uma pessoa que veio até a casa para ver como estamos e se eu me sentia melhor. Alguém que ia ver se eu estava doente, se eu estava bem ou mal e me chamava a atenção” (CF6).
- Consulta para amputados e cuidadores (CF3, CF22, CF27, CF28, CF29, CF32, CF35) – “A gente deveria ter uma consulta no hospital para nos conscientizar mais das coisas e para nos lembrar mais das coisas que devemos fazer, porque tem gente que não tem ideia do trabalho que isso dá, só quem passa por isso” (CF35).
- Suporte telefônico (FC3, FC4, FC5, FC6, FC8, FC14, FC16, FC17, FC18, FC21, FC31, FC33) – “Se houvesse alguém que ligasse, pelo menos eu poderia falar um pouco. Uma pessoa precisa falar sobre nossa tristeza e precisa deixar escapar, mas é isso. Eu não falo muito. No entanto, tudo estava faltando. Não vale a pena sair de casa. Se eles me ligassem para perguntar se estou bem, se não estou bem, eu diria, e é isso. Um telefonema seria suficiente” (CF31).
- Maior tempo de internação (CF33, CF36) – “Eu gostaria de ter tido outro tipo de preparo. Ela deveria ter sido hospitalizada por mais tempo. Então eu poderia me preparar e aprender a fazer as coisas.” (CF36).
- Treinamento de habilidades para cuidadores familiares (CF1, CF3, CF19, CF25, CF28, CF33, CF36, CF39) – “Eu acho que o importante é o treinamento, o fazer. Ver as pessoas fazendo. Era importante para mim que alguém viesse à casa para nos ajudar, treinando-nos. É importante para pessoas que, como eu, por exemplo, não seriam capazes de conhecê-lo...” (CF28).
- Apoio psicológico e mental (CF1, CF3, CF9, CF13, CF18, CF33, CF36) – “A nível psicológico, muda muito, não é? Com o paciente, até mesmo com suas famílias. Por isso é fundamental ter apoio” (CF3).

## DISCUSSÃO

Este estudo explorou o perfil sociodemográfico dos cuidadores familiares de indivíduos com amputação disvascular maior de membros inferiores, com foco naqueles em ambientes de atenção domiciliária. Consoante com pesquisas anteriores, a maioria dos cuidadores eram mulheres (82,5%), que normalmente dedicavam mais horas ao cuidado do que os homens. As mulheres também relataram maiores cargas emocionais e físicas, juntamente com pior saúde psicológica, destacando a disparidade de gênero no cuidado que reforça as desigualdades sociais e limita as oportunidades profissionais^(15,16)^. O estudo também constatou que 65% dos cuidadores tinham mais de 60 anos, sendo o maior grupo (27,5%) com idade entre 66 a 70 anos. Os cuidadores mais velhos enfrentavam maiores desafios financeiros e de saúde, incluindo risco aumentado de doenças crônicas e tempo limitado de autocuidado^(17,18).^ Quase a metade (47,5%) tinha baixa escolaridade e aqueles com letramento em saúde inadequado foram mais comuns entre os cuidadores conjugais do que entre os filhos. Sexo feminino, idade mais avançada e menor escolaridade foram preditores independentes de baixo letramento, o que pode estar relacionado a piores resultados para os receptores de cuidados^(19)^. Além disso, 65% dos cuidadores eram cônjuges ou companheiros e o cuidado conjugal foi associado a relacionamentos conjugais tensos e maior risco de depressão, especialmente durante as transições para dentro ou fora do papel de cuidador^(20,21)^.

Foram destacadas as experiências e as perspectivas dos cuidadores familiares em relação à transição para o papel de cuidador familiar de uma pessoa com amputação maior de membros inferiores de causa vascular. De acordo com a Teoria das Transições de Meleis, os cuidadores familiares passam por mudanças significativas à medida que assumem novos papéis, exigindo o desenvolvimento de novas habilidades e comportamentos, muitas vezes acompanhados de desafios emocionais como perda, isolamento e ansiedade. Essas respostas emocionais são influenciadas pelo apoio social e dificultadas por barreiras como estigma e sobrecarga emocional^(11,22,23)^. Os resultados do presente estudo apoiam essa teoria, com cuidadores relatando sobrecarga substancial, incluindo fadiga, exaustão psicológica e dificuldade em aceitar a amputação, o que contribui para sua tensão emocional. A maioria dos cuidadores eram mulheres com mais de 60 anos e cônjuges, grupos particularmente vulneráveis à sobrecarga do cuidador. Fatores como idade, sexo e status socioeconômico são amplamente reconhecidos por impactar significativamente a saúde mental e as estratégias de enfrentamento^(24)^. As cuidadoras conjugais, em particular, muitas vezes gerenciam múltiplos papéis, além de evitar procurar ajuda e experimentar níveis mais elevados de sobrecarga e sintomas depressivos^(23)^. Os cuidadores também relataram consciência crescente de seu papel e do custo emocional que acarreta, expressando sentimentos mistos de dever, opressão e isolamento. Esses achados destacam a necessidade de estratégias de enfrentamento eficazes para reduzir a sobrecarga do cuidador e melhorar seu bem-estar físico e psicológico^(25,26)^.

Durante a transição para o papel de cuidador familiar, os participantes do estudo destacaram sua consciência de se tornar um cuidador de alguém com amputação maior de membros inferiores de causa vascular. Compreender a resiliência dos membros da família e seu envolvimento no autocuidado é essencial, pois esses fatores podem ajudar a mitigar a sobrecarga do cuidador. Educar os cuidadores sobre a importância do autocuidado é crucial, pois um foco mais forte no autocuidado e na resiliência tem sido associado à redução da sobrecarga do cuidador^(27)^.

O enfermeiro pode apoiar os cuidadores avaliando seu status de transição, oferecendo educação, promovendo apoio social e validando seu papel, transformando a experiência em uma oportunidade de crescimento e resiliência, ao mesmo tempo em que incentiva a adaptação positiva e a confiança em sua nova identidade^(11)^.

Os participantes do presente estudo destacaram as necessidades e as dificuldades que os amputados enfrentam e o impacto do cuidado; relataram a falta de conhecimento da condição de saúde como um problema. A consciência é um aspecto fundamental da transição, evidente em quão bem o conhecimento de um indivíduo sobre o processo alinha-se com suas respostas e percepções durante a transição^(23)^.

A perda de autonomia e mobilidade foi identificada neste estudo pelos cuidadores familiares como uma dificuldade enfrentada pelos amputados maoires de membros inferiores de causa vascular. O principal desafio funcional para amputados de membros inferiores é sua mobilidade limitada na vida diária. O aumento da dependência é consequência dessa restrição^(1)^.

Os cuidadores familiares também relataram que os amputados enfrentam dificuldades relacionadas à adaptação da prótese e ao isolamento social. As restrições de mobilidade experimentadas pelos amputados podem ser atribuídas principalmente ao uso de próteses que não se encaixam adequadamente e ao nível diminuído de confiança dos pacientes em suas próteses. Essa restrição pode levar a maior dependência, reduzindo a acessibilidade ao local de trabalho, à educação, às interações sociais e à vida cotidiana^(1)^.

Outra dificuldade relatada é que os amputados enfrentam medo de cair. As quedas estão ligadas ao medo de cair e à redução da confiança no equilíbrio em pessoas com perda de membros. Essa diminuição da confiança no equilíbrio está associada a níveis mais baixos de função protética^(28)^. Os cuidadores familiares também destacaram a necessidade de apoio psicológico e de saúde mental para os amputados, que devem ser gerenciados durante todo o período de recuperação, mesmo antes da amputação. No entanto, na prática, muitas vezes não recebem apoio ambulatorial de saúde mental até relatarem problemas psicológicos^(29)^.

Uma categoria principal que emergiu deste estudo foi as necessidades/dificuldades do cuidador familiar, com subcategorias relacionadas à necessidade de apoio psicológico e de saúde mental, necessidade de treinamento/informação e de treinamento de habilidades. Os cuidadores precisam de treinamento de habilidades e apoio psicológico e as instituições devem desenvolver serviços para melhorar o apoio ao cuidador, melhorar os resultados educacionais e elevar a qualidade de vida de cuidadores e pacientes, especialmente aqueles com necessidades psicológicas substanciais^(30)^.

Os cuidadores familiares relataram também ter dificuldades com o medo de que a pessoa amputada possa cair. A preocupação com as quedas entre os cuidadores pode ter um impacto prejudicial em seu bem-estar físico e mental, exacerbar a sobrecarga de cuidados e dificultar os esforços para prevenir quedas em casa^(31)^. Esses achados demonstraram que os cuidadores familiares apresentam dificuldades em apoiar as atividades de autocuidado, sendo identificada como um desafio significativo a falta de preparo para o papel. O processo de formação do cuidador informal tem como foco avaliar a capacidade do cuidador de cuidar de alguém com dependência de autocuidado, o que auxilia identificar necessidades específicas de treinamento^(32)^.

Outra categoria principal identificada foi a transição domiciliária. Os participantes descreveram suas experiências de retorno ao domicílio após a alta hospitalar, e subcategorias emergiram relacionadas ao apoio social dos cuidadores familiares que retornam ao domicílio/comunidade: entre elas, destacam-se associações e apoio comunitário, adaptação domiciliar, apoio familiar e de amigos, apoio profissional de saúde e falta de apoio domiciliar. Ter uma rede de apoio é crucial para os cuidadores familiares, pois os ajuda a aliviar a sobrecarga e o estresse do cuidado^(33)^. Os cuidadores podem encontrar alívio e apoio por meio de recursos comunitários, permitindo-lhes atender às diversas necessidades de atendimento ao paciente^(34)^.

Ao fazer a transição domiciliária, os cuidadores familiares referiram que possuíam treinamento de habilidades em relação às atividades de cuidar de um amputado disvascular maior de membro inferior e em casa assumiram os cuidados com o coto para se preparar para a prótese. As avaliações dos cuidadores são essenciais para determinar sua capacidade de assumir papéis e identificar o treinamento necessário. Fornecer educação e treinamento de habilidades pode aumentar a confiança dos cuidadores e melhorar sua capacidade de lidar com os desafios do cuidado diário de forma eficaz^(35)^. A transição do hospital para domicílio após uma grande amputação de membros inferiores devido a doença vascular pode ser emocionalmente desafiadora para pacientes e familiares. Sentir-se informado, envolvido e apoiado é essencial. Programas de cuidados integrados, como a Jornada Segura Dinamarquesa, proporcionam tranquilidade e sensação de segurança para amputados e suas famílias^(36)^.

A última categoria diz respeito a estratégias para promover o empoderamento do cuidador familiar em relação a estratégias que não podem aumentar o empoderamento ao retornar para casa. Tais estratégias aparecem nas seguintes subcategorias: informação e treinamento, serviço de apoio domiciliar, ajudas técnicas, apoio social e econômico, grupos de apoio/pares, livros e informações, visita domiciliária, consulta para amputados e cuidadores, internação mais prolongada, treinamento de habilidades para cuidadores familiares e apoio psicológico e de saúde mental. Esse tipo de estratégia pode ser utilizado na construção de intervenções e programas para promover o empoderamento dos cuidadores familiares de amputados maiores de membros inferiores de causa vascular para o cuidado domiciliário.

A literacia em saúde e a autoeficácia dos cuidadores podem ser melhoradas por meio de intervenções baseadas numa abordagem de empoderamento centrada na família, auxiliando os cuidadores na prestação de cuidados especializados e eficientes e conduzindo a uma melhor qualidade dos cuidados na prestação de cuidados^(37)^. Os cuidadores se beneficiam de maior conhecimento da doença, suas responsabilidades e recursos disponíveis. Depois de atender suas necessidades informacionais, podem ganhar ainda mais com o treinamento e as intervenções de resolução de problemas destinados a gerenciar os comportamentos dos receptores de cuidados e suas próprias respostas emocionais. Intervenções eficazes devem abordar aspectos práticos e emocionais do cuidado^(9)^.

Intervenções domiciliares conduzidas por enfermeiros podem melhorar significativamente a qualidade de vida relacionada à saúde dos cuidadores de pacientes com condições crônicas ou incapacitantes na comunidade^(38)^. Os programas de intervenção podem ajudar a reduzir a sobrecarga do cuidador em diferentes condições de saúde. Intervenções psicoeducativas para cuidadores de pacientes em hemodiálise têm mostrado efeitos positivos tanto na sobrecarga quanto na qualidade de vida^(39)^. Da mesma forma, intervenções educativas, de apoio e psicológicas têm se mostrado eficazes no alívio da sobrecarga entre os cuidadores de pacientes com doença renal crônica^(40)^.

Este estudo apresentou algumas limitações que devem ser consideradas na interpretação dos achados. Primeiro, ele se concentra apenas nos cuidadores familiares, que podem não capturar totalmente as perspectivas de amputados de membros inferiores maiores disvasculares ou profissionais de saúde envolvidos no processo de cuidado. A pesquisa foi realizada em uma única unidade de cirurgia vascular no norte de Portugal, o que pode limitar a generalização dos resultados para outras regiões ou ambientes de saúde. Embora o projeto de pesquisa de doutorado mais amplo inclua contribuições de enfermeiros e amputados, o contexto geográfico e institucional específico da amostra pode não refletir tendências nacionais ou internacionais. Além disso, o uso de entrevistas semiestruturadas, ainda que permita uma exploração aprofundada, pode ter introduzido o viés do entrevistador. Em que pesem os esforços para mitigar o viés, as perspectivas dos pesquisadores podem influenciar a interpretação dos dados, mesmo com vários codificadores. Finalmente, o desenho transversal do estudo limita sua capacidade de tirar conclusões causais.

Pesquisas futuras devem incluir estudos em vários locais com desenhos longitudinais para capturar uma gama mais abrangente de experiências de cuidado. Estudos longitudinais também são cruciais para examinar como os resultados evoluem ao longo do tempo, fornecendo informações sobre o impacto de longo prazo dos cuidados. Expandir o grupo de participantes para incluir cuidadores familiares de diversas origens socioeconômicas e características demográficas variadas fortalecerá ainda mais as evidências e ampliará a relevância dos achados.

Este estudo enfatiza a necessidade de programas e intervenções de empoderamento centrados na família para apoiar os cuidadores familiares de amputados disvasculares maiores de membros inferiores. Para reduzir a sobrecarga do cuidador e promover seu empoderamento, são essenciais caminhos claros para acessar treinamento, apoio comunitário e serviços psicológicos. Os hospitais devem estabelecer sistemas de encaminhamento para o treinamento de cuidadores, oferecer *workshops* (presenciais ou *online*) e fornecer os recursos necessários, como ajudas técnicas.

A coordenação entre hospitais e cuidados primários de saúde é crucial para garantir a continuidade dos cuidados, conectando os cuidadores aos recursos da comunidade, como grupos de apoio, ajuda social e financeira e serviços de atenção domiciliar, com acompanhamento contínuo e apoio acessível. Os serviços psicológicos, incluindo aconselhamento por telefone para cuidadores rurais, também devem ser facilmente acessíveis. Essas iniciativas podem orientar o desenvolvimento de políticas, otimizar a alocação de recursos e garantir que os cuidadores recebam apoio adequado durante a transição do hospital para o domícilio. Os enfermeiros desempenham papel fundamental na educação dos cuidadores sobre cuidados diários, autocuidado, gerenciamento de cotos, coordenação de cuidados e encaminhamento para recursos comunitários, garantindo assim que os cuidadores se sintam capacitados e apoiados.

Para aumentar o valor prático dessas descobertas, é crucial detalhar sua implementação e avaliação. Os profissionais de saúde e os formuladores de políticas podem integrar essas intervenções aos modelos de atendimento, padronizando os processos de encaminhamento, incorporando a educação do cuidador ao planejamento da alta e garantindo recursos comunitários por meio de parcerias formais. O *feedback* regular do cuidador pode ser usado para avaliar a eficácia do treinamento, apoio psicológico e grupos de pares, permitindo melhorias contínuas. Etapas claras para integração e avaliação fortalecerão as aplicações do mundo real das iniciativas de empoderamento do cuidador familiar.

## CONCLUSÃO

Os cuidadores familiares desempenham um papel crucial no apoio aos amputados, fornecendo cuidados emocionais e físicos enquanto gerencia e mudanças significativas no estilo de vida. Geralmente enfrentam isolamento, medo e problemas de saúde mental, o que ressalta a necessidade de priorizar seu bem-estar. Capacitar os cuidadores requer uma abordagem abrangente, incluindo recursos educacionais, treinamento de habilidades, serviços de apoio domiciliário e acesso a apoio social e psicológico. Fortalecer as redes sociais e abordar o bem-estar emocional são essenciais para melhorar a qualidade de vida de cuidadores e amputados.

Esta pesquisa destacou o profundo impacto da amputação maior de membros inferiores de causa vascular nos cuidadores e a necessidade crítica de sistemas de apoio abrangentes para atender suas necessidades em evolução durante a transição do hospital para o domicílio. Os resultados enfatizam a importância de desenvolver programas e intervenções de empoderamento centrados na família que integrem estratégias hospitalares, como treinamento de cuidadores e desenvolvimento de habilidades para tarefas diárias de cuidado e autocuidado, com intervenções baseadas na comunidade, incluindo apoio financeiro, redes de pares e cuidados de saúde mental.

Pesquisas futuras devem explorar a implementação e a eficácia desses programas para avaliar seu potencial para reduzir a sobrecarga dos cuidadores, promover o empoderamento e melhorando os resultados para cuidadores e amputados.

## Data Availability

Os dados deste estudo estão disponíveis mediante solicitação ao autor correspondente.

# Translation: Experiencias y desafíos de cuidadores familiares de personas amputadas de sus miembros inferiores: estudio cualitativo

**DOI:** 10.1590/1980-220X-REEUSP-2024-0264es

**Authors:** Diana Fonseca Rodrigues, António Luís Rodrigues Faria de Carvalho, Cristina Maria Correia Barroso Pinto

**Affiliations:** 1 Universidade do Porto, Faculdade de Medicina, RISE-Health, Porto, Portugal.; 2 Universidade do Porto, Instituto de Ciências Biomédicas Abel Salazar, Porto, Portugal.; 3 Escola Superior de Enfermagem do Porto, Porto, Portugal.

**Keywords:** Amputados, Empoderamiento, Cuidadores, Transición del Hospital al Hogar, Miembro inferior, Investigación Cualitativa

## Abstract

**Objetivo:**

Indagar sobre las experiencias de los cuidadores familiares en transición a su papel de cuidadores de personas con amputación mayor de miembros inferiores de causa vascular para identificar los retos a los que se enfrentan, comprender sus necesidades específicas durante esta transición, examinar sus experiencias durante el traspaso del hospital al hogar y recopilar informaciones sobre estrategias que promueven el empoderamiento de los cuidados.

**Método:**

Estudio descritivo, transversal y exploratorio se llevó a cabo por enfoque cualitativo, mediante entrevistas semiestructuradas entre 40 cuidadores familiares que empezaron a ocuparse de individuos con amputación disvascular mayor de miembros inferiores. Los datos se analizaron con el *software* ATLAS.ti y el análisis del contenido, con el método de Bardin.

**Resultados:**

El análisis del contenido identificó cinco categorías: 1) papel del cuidador familiar, 2) necesidades/dificultades del amputado, 3) necesidades/dificultades del cuidador familiar, 4) transición al hogar y 5) estratégias para promover el empoderamiento del cuidador familiar, con destaque en aspectos críticos de la experiencia de cuidado e identificación de las áreas de intervención.

**Conclusão:**

Los resultados resaltan la necesidad de programas de empoderamiento enfocados en la familia, combinando capacitación hospitalaria con apoyo comunitario. Futuras investigaciones deben sopesar su impacto en la reducción de la sobrecarga del cuidador y en la mejora de los resultados, tanto para los cuidadores como para los amputados.

## INTRODUCCIÓN

La amputación de extremidades supone un reto importante para la salud mundial, ya que afecta profundamente a la vida de las personas y sus familias. Las afecciones vasculares, causadas principalmente por la enfermedad arterial periférica (EAP) y la diabetes mellitus (DM), son las principales causas de amputación de las extremidades inferiores^(1,2)^. Más del 80% de las amputaciones de las extremidades inferiores se deben a causas vasculares, principalmente la enfermedad arterial periférica (EAP), la diabetes mellitus (DM) o una combinación de ambas^(3)^. Los estudios que miden la capacidad funcional 12 meses después de una amputación de causa vascular de miembros inferiores (AMI) han informado de una función física deficiente, con solo el 39% de los pacientes volviendo a su nivel anterior de movilidad^(4)^. Someterse a una amputación de extremidades inferiores es un acontecimiento que altera la vida y puede afectar negativamente a la salud física y mental^(2)^.

Los resultados de la rehabilitación tras una amputación de causa vascular suelen ser deficientes, y las personas afectadas experimentan un nivel de discapacidad superior al 95% en relación con la población general^(5)^. Las amputaciones de miembros inferiores (AMI) pueden dar lugar a limitaciones físicas que pueden restringir las capacidades funcionales de los amputados en las actividades diarias, lo que a menudo conduce a una pérdida significativa de independencia y a una mayor dependencia de los demás^(1)^. Una persona dependiente es aquella que tiene una capacidad limitada o no puede iniciar y llevar a cabo actividades esenciales para su bienestar, salud y mantenimiento de la vida sin la ayuda de otra persona^(6)^. Las personas pueden experimentar situaciones en las que no pueden satisfacer suficientemente sus necesidades de autocuidado, lo que da lugar a déficits de autocuidado. Esto puede deberse a factores como enfermedades, lesiones, discapacidades o falta de conocimientos y recursos^(7)^.

Los cuidadores informales desempeñan un papel crucial en el apoyo a los familiares, ya que les ayudan con las actividades de la vida diaria, realizan tareas médicas y de enfermería, proporcionan apoyo psicosocial y se comunican con los profesionales sanitarios^(8)^. Los cuidadores familiares son familiares, amigos, parejas o vecinos que ofrecen apoyo no remunerado a personas con limitaciones físicas, mentales o cognitivas. La competencia, las habilidades y la motivación de los cuidadores pueden variar por varias razones^(9)^. Estudios anteriores han demostrado que el cuidado de las amputaciones genera estrés psicológico, económico y físico en las familias. La carga del cuidador aumenta significativamente con la gravedad de la amputación, siendo las amputaciones mayores más exigentes que las menores^(10)^. Las tareas del cuidador aumentan progresivamente en intensidad a medida que la discapacidad del receptor de los cuidados empeora, haciéndose más largas, complejas y estresantes. Estas crecientes exigencias pueden conducir a respuestas emocionales negativas, que a su vez pueden desencadenar reacciones físicas o de comportamiento, aumentando el riesgo del cuidador de sufrir problemas de salud mental o física^(9)^.

Un cambio en el estado de salud de un miembro de la familia hará que otros miembros de la familia asuman el papel de cuidador, además de desencadenar una transición dentro de la familia. Un miembro de la familia que esté dispuesto a asumir la responsabilidad de cuidador experimentará una transición situacional, que provocará cambios tanto en su propia vida como en la de la persona dependiente. Aprender y adquirir nuevas habilidades son los componentes esenciales de este proceso^(11)^. Apoyar a los cuidadores informales es esencial no solo para su bienestar, sino también para la sociedad. Destacar la pesada carga y las necesidades a menudo insatisfechas de los cuidadores puede ayudar a la comunidad sanitaria a tomar conciencia del impacto de la amputación en la familia de un paciente^(10)^.

Los cuidadores familiares desempeñan un papel fundamental en los planes de atención de los pacientes con enfermedades crónicas. A medida que aumenta la dependencia de los pacientes de los cuidadores, los enfermeros se enfrentan al reto de garantizar tanto la seguridad del paciente como la calidad de la atención. Al reforzar la comunicación y proporcionar un apoyo sólido, los enfermeros pueden mejorar la competencia de los cuidadores dotándolos de las habilidades necesarias para mantener la seguridad del paciente^(12)^. El personal de enfermería puede poner en marcha intervenciones para capacitar a los cuidadores familiares y mitigar su carga. A diferencia de los modelos educativos tradicionales, el empoderamiento de los cuidadores hace hincapié en la autogestión, transformando la transmisión pasiva de información en una colaboración activa entre cuidadores y pacientes^(13)^.

Este estudio forma parte de un proyecto de investigación doctoral más amplio que explora las perspectivas de las enfermeras y de las personas con amputación mayor de miembro inferior por causa vascular. Su objetivo es contribuir al desarrollo de intervenciones y programas para apoyar a los cuidadores familiares en la gestión del proceso de atención y en el tratamiento de las dimensiones físicas, emocionales y sociales de la prestación de cuidados. Los principales objetivos de este estudio fueron los siguientes:

- Explorar la experiencia de la transición hacia el papel de cuidador familiar de una persona con amputación mayor de miembro inferior de causa vascular.
- Identificar las dificultades y los retos a los que se enfrentan los cuidadores familiares a la hora de cuidar a una persona con amputación mayor de miembro inferior de causa vascular.
- Identificar las necesidades/dificultades de los cuidadores familiares de una persona con amputación mayor de miembro inferior de causa vascular en su transición al papel de cuidador.
- Explorar la experiencia de los cuidadores familiares de una persona con amputación mayor de miembro inferior de causa vascular durante la transición del hospital al hogar.
- Comprender las perspectivas de los cuidadores familiares con respecto a las estrategias para promover su empoderamiento en el cuidado de una persona con amputación mayor de miembro inferior de causa vascular.

## MÉTODO

### Diseño y Configuración del Estudio

Se trata de un estudio exploratorio, transversal, descriptivo y cualitativo realizado en una unidad de cirugía vascular de un hospital del norte de Portugal. Durante el estudio, se consideraron cuidadosamente criterios de investigación rigurosos y se siguieron los Criterios Consolidados para la Presentación de Estudios Cualitativos (COREQ) para la presentación de investigaciones cualitativas. Los datos fueron recopilados entre mayo de 2022 y junio de 2023 por uno de los investigadores con experiencia en la realización de entrevistas que no formaba parte del equipo de la unidad.

### Selección de Participantes

Los participantes fueron seleccionados entre los cuidadores familiares que acompañaron a los pacientes con amputación mayor de miembros inferiores de causa vascular a la consulta hemodinámica de seguimiento de enfermedades vasculares en un servicio de cirugía vascular de un hospital del norte de Portugal.

Los cuidadores familiares fueron seleccionados de acuerdo con los siguientes criterios: (1) ser mayor de 18 años; (2) proporcionar cuidados en el hogar a una persona con amputación mayor de un miembro inferior por enfermedad vascular; (3) ser identificados como cuidadores principales que habían estado cuidando a la persona amputada desde el alta; y (4) cuidar a personas amputadas que requerían asistencia con las actividades de la vida diaria.

La recopilación de datos se llevó a cabo durante un periodo de más de 13 meses, y se centró en toda la población de cuidadores familiares de pacientes con amputaciones mayores de extremidades inferiores debido a enfermedad arterial periférica (EAP) que asistieron a consultas de seguimiento hemodinámico post-amputación para enfermedades vasculares. La selección de los participantes se basó en lograr la saturación de datos, lo que ocurrió después de realizar 40 entrevistas con los cuidadores familiares de personas con amputaciones de causa vascular. La saturación se define como el punto en el que los datos adicionales ya no pueden contribuir a nuevos conocimientos sobre el fenómeno en estudio, lo que conduce a la redundancia. Según Bardin^(14)^, los investigadores deben estar atentos durante la categorización y el análisis para reconocer cuándo los datos dejan de aportar nuevos elementos, lo que marca el logro de la saturación, que se confirmó en este estudio después de 40 entrevistas.

En este estudio cualitativo, se identificaron posibles sesgos y se tomaron medidas para mitigarlos. Una de las principales preocupaciones era el sesgo de selección, ya que los participantes podrían haber sido seleccionados en función de su disponibilidad o voluntad de participar. Para minimizar este sesgo, se hizo lo posible por incluir a personas de diferentes grupos de edad y entornos socioeconómicos. Aunque el reclutamiento no se llevó a cabo en múltiples contextos, este enfoque ayudó a garantizar una muestra más variada y representativa de estos cuidadores familiares.

### Recogida de Datos

Los datos se recogieron mediante un cuestionario sociodemográfico desarrollado para este estudio con variables sociodemográficas de género, edad, nivel educativo y situación laboral en el momento de la entrevista. Se realizaron entrevistas semiestructuradas utilizando un guion con preguntas sobre las experiencias de los cuidadores en relación con la prestación de cuidados y apoyo a una persona con una amputación mayor de una extremidad inferior debido a una enfermedad vascular después del alta hospitalaria y el regreso a casa (Cuadro 1).

**Cuadro 1:** Guión de preguntas – Vila Nova de Gaia, VNG, Portugal, 2022–2023.

Como cuidador familiar de un paciente con una amputación de miembro inferior, ¿cómo se siente? ¿Qué cambios se han producido en su vida desde que asumió el papel de cuidador? ¿Puede decirme cómo se preparó para asumir este papel y proporcionar cuidados? ¿Qué dificultades ha experimentado? ¿Sigue enfrentándose a dificultades ahora? Si es así, ¿cuáles son? Cuando su familiar regresó a casa, ¿quién le ayudó a superar los retos a los que se enfrentaba? ¿Cree que la preparación que recibió durante la estancia en el hospital fue suficiente o habría preferido una formación diferente? Al cuidar de su familiar, ¿siente la necesidad de recibir más formación y apoyo? ¿Qué considera importante y qué debería mejorarse para ayudar a las personas en su situación a superar los retos a los que se enfrentó al volver a casa con una persona dependiente?

El investigador principal programó visitas a domicilio para realizar entrevistas individuales con el cuidador familiar. Después de obtener la firma del formulario de consentimiento informado, las entrevistas se grabaron en audio digital y posteriormente se transcribieron. Cada sesión duró aproximadamente entre 20 y 25 minutos. La recopilación de datos continuó hasta que se alcanzó la saturación de datos.

### Análisis y Tratamiento de Datos

Se realizó un análisis cualitativo de contenido utilizando la metodología de Bardin^(14)^ y el software ATLAS.ti 23.3.4 (Thomas Muhr, Berlín, Alemania). El anonimato de los participantes se protegió mediante un sistema de codificación que consistía en dos letras seguidas de un número.

El enfoque temático-categórico comprende tres fases principales. En la **fase de preanálisis**, los investigadores realizaron una lectura flotante de las transcripciones para obtener una comprensión general e identificar categorías preliminares, sentando las bases para análisis posteriores. La **exploración del material** implicó la organización de datos dentro de ATLAS.ti, donde los investigadores crearon códigos que representaban unidades de significado y los agruparon en categorías temáticas. El software facilitó la visualización clara de las relaciones entre códigos y la comprensión de las frecuencias de los temas, mejorando la comprensión del contenido. En el **tratamiento de los resultados y la fase de** interpretación, los investigadores hicieron inferencias basadas en un marco teórico.

Cada fase de análisis fue realizada de forma independiente por dos investigadores, con un tercero consultado en caso de discrepancias. En última instancia, a través del análisis y la discusión, los investigadores llegaron a un consenso sobre los resultados. La integración de la metodología de Bardin^(14)^ con ATLAS.ti permite un análisis estructurado y exhaustivo, que conduce a hallazgos significativos y bien fundamentados.

Se tuvo en cuenta el sesgo de los investigadores, ya que las percepciones personales podrían influir en la interpretación de los datos. Para minimizarlo, el análisis temático fue realizado por varios investigadores que codificaron los datos de forma colaborativa. Este enfoque aseguró la triangulación y facilitó las discusiones colectivas de varias interpretaciones, fortaleciendo en última instancia la validez de los hallazgos.

### Aspectos Éticos

La investigación siguió los principios deontológicos recomendados por el Comité de Ética para el Estudio de Sujetos Humanos. El comité de ética del hospital aprobó el estudio y garantizó el cumplimiento de las directrices de la Declaración de Helsinki en materia de autodeterminación, privacidad, anonimato, confidencialidad y consentimiento informado. Se preservó el anonimato de los participantes asignándoles letras y números (CF = Cuidador Familiar, junto con un número de entrevista secuencial).

## RESULTADOS

### Caracterización de los Participantes

Los participantes en el estudio eran cuidadores principales que eran familiares y tenían experiencia previa en el cuidado de personas con amputación mayor de miembros inferiores de causa vascular, que necesitaban ayuda de cuidadores familiares con las actividades de la vida diaria y que vivían en casa. En el estudio participaron 40 personas, de las cuales el 82,5% eran mujeres. La mayoría de los cuidadores familiares tenían más de 60 años, lo que representaba el 65% del grupo. Los cuidadores de entre 66 y 70 años constituían la mayor proporción, representando el 27,5% de los participantes. Teniendo en cuenta el nivel educativo, el 47,5% tenía cuatro años de educación básica; en el momento del estudio, el 37,5% de los participantes eran empleados activos. En cuanto a la relación entre los cuidadores familiares y las personas amputadas, el 65% de ellos eran cónyuges o parejas.

Del análisis de contenido de las entrevistas surgieron cinco subcategorías: 1) rol del cuidador familiar, 2) necesidades/dificultades del amputado, 3) necesidades/dificultades del cuidador familiar, 4) transición al hogar y 5) estrategias para promover el empoderamiento del cuidador familiar. Las categorías y subcategorías identificadas en el análisis de contenido de las entrevistas se resumen en la Cuadro 2.

**Cuadro 2:** Categorías y subcategorías surgidas de las entrevistas – Vila Nova de Gaia, VNG, Portugal, 2022–2023.

Categorías	Subcategorías
**Rol del cuidador familiar**	– Carga del cuidador familiar – Cambios en el estilo de vida – Aceptación de la amputación – Conciencia del rol del cuidador familiar – Sentimientos y actitudes de los cuidadores
**Necesidades/dificultades del amputado**	– Pérdida de autonomía y movilidad – Conciencia sobre la condición de salud – Aislamiento social – Necesidad de apoyo psicológico y de salud mental – Miedo a caídas – Adaptación a la prótesis
**Necesidades/dificultades del cuidador familiar**	– Necesidad de apoyo psicológico y de salud mental – Miedo del cuidador a las caídas – Falta de preparación para el rol – Autocuidado – Necesidad de formación e información – Necesidad de entrenamiento en habilidades
**Transición al hogar**	– Apoyo de asociaciones y comunidades – Adaptación del hogar – Cuidado del muñón – Capacitación para cuidadores familiares – Apoyo de familiares y amigos – Apoyo de profesionales de la salud – Falta de apoyo en el hogar
**Estrategias para fortalecer al cuidador familiar**	– Información y formación – Servicio de apoyo en el hogar – Ayudas técnicas – Apoyo social y económico – Grupos de apoyo/pares – Libros y folletos informativos – Visitas domiciliarias – Consulta para amputados y cuidadores – Apoyo telefónico – Hospitalización prolongada – Capacitación para cuidadores familiares – Apoyo psicológico y de salud mental

### El Papel del Cuidador Familiar

Esta categoría se centra en las experiencias y perspectivas de los cuidadores familiares que se encargan de atender a personas con amputación de miembros inferiores de causa vascular. Esta categoría incluye las siguientes subcategorías.

- Carga del cuidador familiar (CF1, CF3, CF4, CF6, CF11, CF15, CF17, CF19, CF21, CF31, CF33, CF35) - «Psicológicamente, esto es agotador. Esto fue estresante y agotador. Me considero una persona fuerte. Sin embargo, esto es muy difícil en el primer año. Sin embargo, hay muchas dificultades. A nivel psicológico, te entristeces. Algunos días estamos de bajón. Estamos cansados. Porque tengo que cuidarme en este momento» (CF19).
- Cambios en el estilo de vida (CF2, CF4, CF7, CF10, CF14, CF16, CF17, CF18, CF19, CF20, CF23, CF26, CF28, CF31, CF32, CF36, CF38, CF39) - «Los cambios, en mi vida lo eran todo. Ella solía hacerlo todo: ahora soy yo. Debo hacerlo todo, así que limpiar la casa, limpiar a mi mujer, mi higiene personal, preparar la comida, las cenas, los aperitivos, todo, lo hago todo» (CF26).
- Aceptar la amputación (CF5, CF8, CF16, CF22, CF33, CF34) – «Es mucho amor por la propia persona. Me gustó mucho, mucho. Después de ver eso, no fue la falta de una pierna. En este caso, se amputó la pierna del paciente. No es la falta de una pierna porque lo miro, como siempre lo he mirado. Nunca sentí que le faltara una pierna» (CF22).
- Conciencia del papel de cuidador familiar (CF9, CF14, CF18, CF33, CF37, CF40): «Siento la obligación de cuidarlo, de ayudarlo, eso es lo que hago. Yo lo ayudaría, y él también me ayudaría mucho. Pero ahora me necesita para quitarme su prótesis, ponérmela, lavarla y ayudarle a bañarse» (CF37).
- Sentimientos y actitudes de los cuidadores familiares (CF2, CF9, CF12, CF13, CF18, CF22, CF24, CF26, CF27, CF28, CF29, CF33, CF34, CF36, CF38, CF39): «¿Entonces el sentimiento es amor? ¿dolor? ¿placer? No lo sé; es una mezcla que debemos hacer, pero no por obligación, sino por voluntad propia. Tengo sentimientos encontrados sobre esta situación. Es mucho» (CF28).

### Necesidades/Dificultades de los Amputados

La segunda categoría surgió de las perspectivas y experiencias de los cuidadores familiares en relación con el cuidado de la persona con amputación mayor de miembro inferior de causa vascular y las necesidades y dificultades a las que se enfrentan después de la amputación y al regresar a casa. En esta categoría se desarrollan las siguientes subcategorías:

- Pérdida de autonomía y movilidad (CF3, CF29) - «Era muy independiente, muy activo, ayudaba a la gente, era responsable del cementerio y decoraba la tumba de mi madre. No paraba, siempre tenía iniciativa, siempre, siempre, y se iba a las mareas, a los pulpos, a las navajas, siempre caminaba, no paraba; es decir, tuvo mala suerte. Dejó de ser independiente y de hacer cosas» (CF29).
- Conciencia del estado de salud (CF22): «Luego estaba el problema de que el amputado olvida que es un amputado, ¿verdad? Se levanta y hay situaciones así. Se cayó cuando salió del hospital, cuando le abrieron el muñón, e incluso más tarde, tuvo que ir al hospital para que le arreglaran algunos de los puntos».
- Aislamiento social (CF21, CF22, CF29, CF38) – «Mi marido está aislado en casa, porque la casa no tiene ascensor, y solo sale con los bomberos para ir a las citas» (CF38).
- Necesidad de apoyo psicológico y mental (CF11, CF19, CF20, CF33, CF39) - «Si es a nivel psiquiátrico, para el propio paciente. Pienso en los principios para probar, o no sé, un psiquiatra, o algo así, porque en el caso del dolor fantasma. Sé que una noche o dos, una vez me dijo que soñó que tenía el pie» (CF20).
- Miedo a caerse (CF34) - «Tiene miedo a caerse, porque cuando se encuentra mejor, va al campo, pero ya se ha caído dos veces, así que tiene miedo, porque el médico le había dicho que tenía que tener mucho cuidado, porque si se cae, no sé cómo va a ser después» (CF34).
- Colocación de la prótesis (CF19) - «El médico rehabilitador no lo vio para saber si estaba bien con la prótesis, si se ajustaba bien, si tenía que hacer cambios, nada. Solo le recetó una prótesis que no era compatible con él, y el servicio nacional de salud gastó bastante, no sé cuántos miles de euros, porque eso es caro, seguro que más de tres mil euros. El sistema nacional de salud gastó este dinero para que esto se dejara de lado».

### Necesidades/Dificultades de los Cuidadores Familiares

Esta categoría describe las necesidades y dificultades que experimentan los cuidadores familiares cuando atienden a una persona con amputación inferior mayor de causa vascular que vive en casa. Esta categoría incluye las siguientes subcategorías.

- Necesidad de apoyo psicológico y de salud mental (CF18, CF29, CF39, CF40): «Fue muy malo. Puedo decir que esta fue una de las peores fases de mi vida. Nunca habíamos estado tan conmocionados. Me faltaba apoyo y me sentía muy sola. Fui a ver a un psicólogo para que me apoyara en esta situación que me estaba pasando» (CF40).
- Miedo a caerse de los cuidadores familiares (CF17, CF23, CF31, CF33, CF35, CF38): «Solo tenía miedo de que si lo levantaba, se me cayera encima. A veces se caía y se le abrían algunos de los puntos, etc. Era mi miedo. Mi única dificultad era el miedo a que se me cayera encima» (CF17).
- Falta de preparación para el papel (CF5, CF6, CF9, CF13, CF18, CF34, CF39, CF40): «Creo que fue un poco de falta de comunicación, alguien podría llamarnos y decirnos algo como: mira, vas a hacer esto, porque tienes que comprar esto, o tienes que comprar aquello, o la seguridad social ayuda con esto» (CF9).
- Cuidado personal (CF3, CF5, CF6, CF12, CF10, CF14, CF24, CF25, CF26, CF27, CF28, CF30, CF31, CF32, CF34, CF35, CF38): «Me costaba bañarlo porque era amputado. La falta de equilibrio y todo era un problema, más tarde compramos un asiento adecuado para meterlo en la ducha» (CF34).
- Necesidad de formación/información (CF1, CF5, CF10, CF11, CF16, CF17, CF18, CF28, CF36, CF39) – «Creo que hace falta más información, informar a la gente, decirles que tienen derecho a hacer esto, que tienen derecho a esta ayuda y que pueden solicitarla más. Más conocimientos o más formación» (CF1).
- Necesidad de formación en habilidades (CF29): «Me gustaría que la enfermera me enseñara a hacerlo porque él siempre será un amputado. Incluso en lo que respecta al baño, aunque sea una persona encamada. Hay cosas, hay pequeños trucos que no sabemos porque en el momento en que lo llevé, venía con un pañal, la persona se giraba hacia un lado, le ponía el pañal y se giraba hacia el otro, cosas así, como vimos».

### Transición al Hogar

La presente categoría describe las experiencias de los cuidadores familiares en la provisión de cuidados durante la transición al hogar después del alta de una persona con amputación mayor de miembro inferior de causa vascular. En esta categoría surgieron las siguientes subcategorías:

- Asociación y apoyo comunitario (CF35): «Así es, la gente del centro de día viene a ayudarnos a bañarlo, de domingo a domingo. Yo soy quien paga con mi dinero, y lo quiero de domingo a domingo porque la higiene es muy importante. Yo tampoco podría bañarla sola».
- Adaptación del hogar (CF1, CF12, CF14, CF16, CF19, CF21, CF27, CF35, CF38) - «Fue adaptar la habitación, adaptar el baño y darle lo mejor, porque hemos gastado mucho dinero y solo somos dos jubilados...» (CF27).
- Cuidado del muñón (CF11) – «Pero antes de tener la prótesis, había que vendarlo, con un vendaje, en el que había que vendar todo el muñón, porque estaba más recto, para que no se abriera tanto o se volviera demasiado plano».
- Formación de habilidades para cuidadores familiares (CF31): «Fui al hospital para aprender un poco sobre cómo tratar con él, en la cama, y nada más. Fui unas cuantas veces. Fui unas cuantas veces. Aprendí a darle la vuelta en la cama y ponerle el pañal, pero claro, él debe tener fuerza, pero ha cambiado.
- Apoyo de familiares y amigos (CF1, CF2, CF4, CF6, CF8, CF9, CF11, CF12, CF13, CF16, CF18, CF19, CF20, CF23, CF24, CF25, CF28, CF30, CF33, CF34, CF35, CF36, CF39, CF40) - «Mi hija y yo, y el resto era yo. Solo el núcleo familiar me ayudó; no tuve más ayuda. Mi hija y yo nos miramos, y también nos ayudó mi yerno» (CF28).
- Apoyo de los profesionales sanitarios (CF13): «Sabía que podía contar con mi fisioterapeuta, que siempre me apoyó mucho. Me habló de las transferencias, de cómo estaba realmente, de cómo se hacía, y de cómo se hacía. Básicamente, reforzó lo que yo estaba empezando a ver».
- Falta de apoyo en el hogar (CF34, CF39): «Nadie me preguntó nunca, por ejemplo, en el hospital si necesitaba seguimiento; si necesitaba hablar con alguien, nadie acudió a mí; acudí porque sentí esa necesidad, y eso es, fui por mi cuenta» (CF39).

### Estrategias Para Promover el Empoderamiento de los Cuidadores Familiares

Esta categoría surgió de las experiencias y perspectivas de los cuidadores familiares con respecto a las estrategias para promover el empoderamiento en el cuidado de una persona con amputación mayor de la extremidad inferior de causa vascular. Las subcategorías son las siguientes.

- Información y formación (CF1, CF3, CF8, CF9, CF24, CF26, CF27, CF29) - (...) Podría haber habido información más detallada, creo que podría haberla habido. Podrían haberme dado alguna información de una enfermera o un médico, posiblemente, o podrían haberme preguntado si era capaz de hacer esto o aquello, y eso debería haberse hecho (CF8).
- Servicio de asistencia a domicilio (CF13, CF31, CF32, CF33, CF34, CF40): «Si alguien viniera a casa y me dijera: haz esto o aquello, siempre sería bueno. Incluso podría estar haciendo algo y aprender a hacerlo más fácilmente si tuviera a alguien que me lo explicara. Era necesario que alguien se presentara en casa» (CF32).
- Ayudas técnicas (CF1, CF39): «Por ejemplo, si no tienes una cama articulada y no puedes permitírtela, alguien debería proporcionártela; si no tienes una silla de ruedas y la necesitas, alguien debería conseguirte una» (CF1).
- Apoyo social y económico (CF15, CF26) - «En otras palabras, si no hay un servicio social, al menos debería haber una prestación económica, en la que la gente pueda pagar a alguien para que le ayude a cuidar del amputado» (CF15).
- Apoyo/grupo de pares (CF39) – «También formé parte de un grupo de familiares de personas que también era un grupo universitario (...) Está bien porque a veces este intercambio entre personas que comparten las mismas dificultades y retos, creo que nos ayuda a encajar y a resolver fácilmente algunos problemas».
- Libros y folletos informativos (CF1, CF3, CF13): «Algunos folletos pequeños, libritos para llevar a casa con una imagen, con todo muy atractivo, eso siempre funciona muy bien» (CF13).
- Visita a domicilio (CF1, CF4, CF5, CF6, CF11, CF12, CF14, CF16, CF17, CF19, CF20, CF21, CF23, CF31, CF34, CF35, CF39, CF40): «Deberíamos contar con el apoyo de una persona que viniera a casa para ver cómo estamos y si me siento mejor. Alguien que fuera y viera si estaba enfermo, si me encontraba bien o mal y me llamara la atención» (CF6).
- Consulta para amputados y cuidadores (CF3, CF22, CF27, CF28, CF29, CF32, CF35): «Deberíamos tener una cita en el hospital, para ser más conscientes de las cosas y para que nos recuerden más las cosas que debemos hacer, porque hay gente que no tiene ni idea del trabajo que esto supone, solo los que lo pasan» (CF35).
- Asistencia telefónica (CF3, CF4, CF5, CF6, CF8, CF14, CF16, CF17, CF18, CF21, CF31, CF33): «Si llamara alguien, al menos podría hablar un poco. Una persona necesita hablar de su tristeza y necesita desahogarse, pero eso es todo. Yo no hablo mucho. Sin embargo, faltaba todo. No merece la pena salir de casa. Si me llamaran para preguntarme si estoy bien, si no estoy bien, lo diría, y ya está. Una llamada telefónica sería suficiente» (CF31).
- Hospitalización más prolongada (CF33, CF36): «Me hubiera gustado haber tenido otro tipo de preparación. Debería haber estado hospitalizada durante más tiempo. Así, podría haberme preparado y haber aprendido a hacer las cosas» (CF36).
- Formación de habilidades para cuidadores familiares (CF1, CF3, CF19, CF25, CF28, CF33, CF36, CF39) – «Creo que lo importante es la formación, el hacer. Ver a la gente haciendo. Para mí era importante que alguien viniera a casa a ayudarnos formándonos. Es importante para la gente que, como yo por ejemplo, no podría saberlo...» (CF28).
- Apoyo psicológico y mental (CF1, CF3, CF9, CF13, CF18, CF33, CF36) – «A nivel psicológico, cambia mucho, ¿no? Con el paciente, incluso con sus familias. Por eso es esencial contar con apoyo» (CF3).

## DISCUSIÓN

Este estudio explora el perfil sociodemográfico de los cuidadores familiares de personas con amputación mayor de miembro inferior por enfermedad vascular, centrándose en aquellos en entornos de atención domiciliaria. En consonancia con investigaciones anteriores, la mayoría de los cuidadores eran mujeres (82,5%), que normalmente dedicaban más horas a la prestación de cuidados que los hombres. Las mujeres también refieren mayores cargas emocionales y físicas, junto con una peor salud psicológica, lo que pone de relieve la disparidad de género en la prestación de cuidados que refuerza las desigualdades sociales y limita las oportunidades profesionales^(15,16)^. El estudio también reveló que el 65% de los cuidadores tenían más de 60 años, siendo el grupo más numeroso (27,5%) con edades entre 66 y 70 años. Los cuidadores de más edad se enfrentan a mayores dificultades sanitarias y económicas, como un mayor riesgo de enfermedades crónicas y un tiempo limitado para el cuidado personal^(17,18)^. Casi la mitad (47,5%) tenía un nivel educativo bajo, y aquellos con conocimientos sanitarios inadecuados eran más comunes entre los cónyuges cuidadores que entre los hijos. El sexo femenino, la edad avanzada y el nivel educativo más bajo fueron predictores independientes de un bajo nivel de alfabetización en salud, lo que puede estar relacionado con peores resultados para los beneficiarios de los cuidados^(19)^. Además, el 65% de los cuidadores eran cónyuges o parejas, y el cuidado del cónyuge se asoció con relaciones matrimoniales tensas y un mayor riesgo de depresión, especialmente durante las transiciones hacia o desde el papel de cuidador^(20,21)^).

Los resultados de este estudio destacan las experiencias y perspectivas de los cuidadores familiares con respecto a su transición al papel de cuidador familiar de una persona con amputación mayor de la extremidad inferior de causa vascular. Según la Teoría de las Transiciones de Meleis, los cuidadores familiares experimentan cambios significativos a medida que asumen nuevos roles, lo que requiere el desarrollo de nuevas habilidades y comportamientos, a menudo acompañados de desafíos emocionales como la pérdida, el aislamiento y la ansiedad. Estas respuestas emocionales se ven influidas por el apoyo social y obstaculizadas por barreras como el estigma y la sobrecarga emocional^(11,22,23)^. Nuestro estudio respalda esta teoría, ya que los cuidadores informan de una carga sustancial, que incluye fatiga, agotamiento psicológico y dificultad para aceptar la amputación, todo lo cual contribuye a su tensión emocional. La mayoría de los cuidadores de nuestro estudio eran mujeres mayores de 60 años y cónyuges, grupos especialmente vulnerables a la carga de los cuidadores. Se reconoce ampliamente que factores como la edad, el género y la situación socioeconómica afectan significativamente a la salud mental y a las estrategias de enfrentamiento^(24)^. Las mujeres cuidadoras de cónyuges, en particular, suelen desempeñar múltiples funciones, además de evitar buscar ayuda, y experimentan mayores niveles de carga y síntomas depresivos^(23)^. Los cuidadores también informaron de una creciente conciencia de su función y del coste emocional que conlleva, expresando sentimientos encontrados de deber, agobio y aislamiento. Estos hallazgos ponen de relieve la necesidad de estrategias de enfrentamiento eficaces para reducir la carga del cuidador y mejorar el bienestar físico y psicológico^(25,26)^.

Durante la transición al papel de cuidador familiar, los participantes de nuestro estudio destacaron su conciencia de convertirse en cuidadores de alguien con una amputación mayor de la extremidad inferior por enfermedad vascular. Comprender la resiliencia de los miembros de la familia y su compromiso con el autocuidado es esencial, ya que estos factores pueden ayudar a mitigar la carga del cuidador. Es fundamental educar a los cuidadores sobre la importancia del autocuidado, ya que un mayor énfasis en el autocuidado y la resiliencia se ha asociado con una reducción de la carga del cuidador^(27)^.

Las enfermeras pueden apoyar a los cuidadores evaluando su estado de transición, ofreciendo educación, fomentando el apoyo social y validando su papel, convirtiendo la experiencia en una oportunidad de crecimiento y resiliencia, al tiempo que fomentan la adaptación positiva y la confianza en su nueva identidad^(11)^.

Los participantes en el presente estudio destacaron las necesidades y dificultades a las que se enfrentan las personas amputadas y el impacto de los cuidados; informaron de la falta de concienciación sobre el estado de salud como un problema. La concienciación es un aspecto clave de la transición, evidente en la medida en que el conocimiento que tiene una persona del proceso se alinea con sus respuestas y percepciones durante la transición^(23)^.

En este estudio, los cuidadores familiares identificaron la pérdida de autonomía y movilidad como una dificultad a la que se enfrentan las personas con amputaciones mayores de miembros inferiores por causas vasculares. El principal desafío funcional para las personas con amputación de miembros inferiores es su movilidad limitada en la vida diaria. El aumento de la dependencia es una consecuencia de esta restricción^(1)^.

Los cuidadores familiares también informaron de que las personas con amputación se enfrentan a dificultades relacionadas con el ajuste de las prótesis y el aislamiento social. Las limitaciones de movilidad que experimentan las personas con amputación pueden atribuirse principalmente al uso de prótesis que no se ajustan correctamente y a un menor nivel de confianza en su prótesis. Esta restricción puede conducir a un mayor nivel de dependencia, lo que en última instancia reduce la accesibilidad al lugar de trabajo, la educación, las interacciones sociales y la vida diaria^(1)^. Los participantes también destacaron de que otra dificultad a la que se enfrentan los amputados es el miedo a caerse. Las caídas están relacionadas con el miedo a caerse y la reducción de la confianza en el equilibrio en las personas con pérdida de extremidades. Esta disminución de la confianza en el equilibrio está asociada a niveles más bajos de función protésica^(28)^. Los cuidadores familiares también destacaron la necesidad de apoyo psicológico y de salud mental para los amputados. La salud mental de los amputados debe gestionarse durante todo su período de recuperación, incluso antes de la amputación. Sin embargo, en la práctica, a menudo no reciben apoyo de salud mental ambulatorio hasta que informan de problemas psicológicos^(29)^.

Una categoría principal que surgió de nuestro estudio fueron las necesidades/dificultades de los cuidadores familiares, con subcategorías relacionadas con la necesidad de apoyo psicológico y de salud mental, la necesidad de formación/información y la necesidad de formación en habilidades. Los cuidadores necesitan formación y apoyo psicológico, y las instituciones deben desarrollar servicios para mejorar el apoyo a los cuidadores, mejorar los resultados educativos y elevar la calidad de vida tanto de los cuidadores como de los pacientes, especialmente aquellos con necesidades psicológicas importantes^(30)^.

Los cuidadores familiares informaron de que tenían dificultades con el miedo a caerse. La preocupación por las caídas entre los cuidadores puede tener un impacto perjudicial en su bienestar físico y mental, exacerbar la carga de los cuidados y obstaculizar los esfuerzos para prevenir las caídas en el hogar^(31)^. Los resultados del estudio demostraron que los cuidadores familiares experimentan dificultades para apoyar las actividades de autocuidado, y la falta de preparación para el papel se identificó como un desafío importante. El proceso de formación de cuidadores informales se centra en evaluar la capacidad del cuidador para atender a alguien con dependencia de autocuidado, lo que ayuda a identificar necesidades específicas de formación^(32)^.

Otra categoría principal identificada en nuestro estudio fue la transición al hogar. Los participantes describieron sus experiencias al regresar al hogar después del alta hospitalaria y surgieron subcategorías relacionadas con el apoyo social disponible para los cuidadores familiares que regresan al hogar/comunidad: estas incluyeron el apoyo de la asociación y la comunidad, adaptación del hogar, apoyo de familiares y amigos, apoyo de profesionales de la salud y falta de apoyo en el hogar. Contar con una red de apoyo es crucial para los cuidadores familiares, ya que ayuda a aliviar la carga y el estrés de la prestación de cuidados^(33)^. Los cuidadores pueden encontrar alivio y apoyo a través de los recursos comunitarios, lo que les permite satisfacer las diversas necesidades de la atención al paciente^(34)^.

Al hacer la transición a casa, los cuidadores familiares refirieron que habían recibido capacitación en habilidades relacionadas con las actividades de cuidado de un paciente con amputación mayor de miembro inferior por enfermedad vascular y que en casa asumían el cuidado del muñón para preparar la prótesis. Las evaluaciones de los cuidadores son esenciales para determinar su capacidad para asumir funciones e identificar la formación necesaria. Proporcionar educación y formación puede aumentar la confianza de los cuidadores y mejorar su capacidad para afrontar eficazmente los retos del cuidado diario^(35)^. La transición del hospital al hogar después de una amputación mayor de una extremidad inferior debido a una enfermedad vascular puede ser un reto emocional para los pacientes y sus familias. Es esencial sentirse informado, involucrado y apoyado. Los programas de atención integrada, como el Danish Safe Journey (Jornada Segura dinamarquesa), proporcionan tranquilidad y sensación de seguridad a los amputados y sus familias^(36)^.

La última categoría que surgió de nuestros hallazgos se refería a las estrategias para promover el empoderamiento de los cuidadores familiares en relación con las estrategias que no pueden mejorar el empoderamiento al regresar a casa. Estas estrategias aparecen en las siguientes subcategorías: información y formación, servicio de apoyo a domicilio, ayudas técnicas, apoyo social y económico, grupos de apoyo/entre iguales, libros e información, visita a domicilio, consulta para amputados y cuidadores, hospitalización más prolongada, formación de habilidades para cuidadores familiares y apoyo psicológico y de salud mental. Este tipo de estrategia puede utilizarse en la elaboración de intervenciones y programas para promover el empoderamiento de los cuidadores familiares de personas con amputaciones mayores de miembros inferiores de causa vascular en su transición a la atención domiciliaria.

La alfabetización sanitaria y la autoeficacia de los cuidadores pueden mejorarse mediante intervenciones basadas en un enfoque de empoderamiento centrado en la familia, que ayude a los cuidadores a proporcionar una atención especializada y eficiente y que conduzca a una mejor calidad de la atención^(37)^. Los cuidadores se benefician de un mayor conocimiento de la enfermedad, de sus responsabilidades y de los recursos disponibles. Tras abordar sus necesidades de información, pueden beneficiarse aún más de la formación en resolución de problemas y de las intervenciones destinadas a gestionar los comportamientos de los receptores de cuidados y sus propias respuestas emocionales. Las intervenciones eficaces deben abordar tanto los aspectos prácticos como los emocionales del cuidado^(9)^.

Las intervenciones domiciliarias dirigidas por enfermeras pueden mejorar significativamente la calidad de vida relacionada con la salud de los cuidadores de pacientes con enfermedades crónicas o discapacitantes en la comunidad^(38)^. Los programas de intervención pueden ayudar a reducir la carga de los cuidadores en diferentes condiciones de salud. Las intervenciones psicoeducativas para los cuidadores de pacientes en hemodiálisis han demostrado tener efectos positivos tanto en la carga como en la calidad de vida^(39)^. Del mismo modo, las intervenciones educativas, de apoyo y psicológicas han demostrado su eficacia para aliviar la carga de los cuidadores de pacientes con enfermedad renal crónica^(40)^.

Este estudio tenía algunas limitaciones que deben tenerse en cuenta al interpretar los resultados. En primer lugar, se centra únicamente en los cuidadores familiares, lo que puede no reflejar plenamente las perspectivas de las personas con amputaciones mayores de las extremidades inferiores por problemas vasculares o de los profesionales sanitarios que participan en el proceso de cuidado. La investigación se llevó a cabo en una sola unidad de cirugía vascular en el norte de Portugal, lo que puede limitar la generalización de los resultados a otras regiones o entornos sanitarios. Si bien el proyecto de investigación doctoral más amplio incluye aportaciones de enfermeras y amputados, el contexto geográfico e institucional específico de la muestra puede no reflejar las tendencias nacionales o internacionales. Además, el uso de entrevistas semiestructuradas, aunque permite una exploración en profundidad, puede haber introducido un sesgo del entrevistador. A pesar de los esfuerzos por mitigar el sesgo, las perspectivas de los investigadores pueden influir en la interpretación de los datos, incluso con múltiples codificadores. Por último, el diseño transversal del estudio limita su capacidad para extraer conclusiones causales.

Las investigaciones futuras deberían incluir estudios en múltiples sitios con diseños longitudinales para captar una gama más completa de experiencias de cuidado. Los estudios longitudinales también son cruciales para examinar cómo evolucionan los resultados a lo largo del tiempo, proporcionando información sobre el impacto a largo plazo del cuidado. Ampliar el grupo de participantes para incluir a cuidadores familiares de diversos orígenes socioeconómicos y características demográficas variadas reforzará aún más las pruebas y ampliará la relevancia de nuestros hallazgos.

Este estudio enfatiza la necesidad de programas e intervenciones de empoderamiento centrados en la familia para apoyar a los cuidadores familiares de personas con amputaciones mayores de miembros inferiores por enfermedad vascular. Para reducir la carga del cuidador y promover el empoderamiento, es esencial contar con vías claras para acceder a la formación, el apoyo comunitario y los servicios psicológicos. Los hospitales deben establecer sistemas de derivación para la formación de cuidadores, ofrecer talleres (en línea o presenciales) y proporcionar los recursos necesarios, como ayudas técnicas.

La coordinación entre los hospitales y la atención primaria de salud es crucial para garantizar la continuidad de la atención, conectando a los cuidadores con los recursos comunitarios, como grupos de apoyo, ayuda social y financiera, y servicios de atención domiciliaria, con un seguimiento continuo y un apoyo accesible. Los servicios psicológicos, incluida la orientación telefónica para los cuidadores rurales, también deben ser fácilmente accesibles. Estas iniciativas pueden orientar la elaboración de políticas, optimizar la asignación de recursos y garantizar que los cuidadores reciban el apoyo adecuado durante la transición del hospital al hogar. Las enfermeras desempeñan un papel clave en la educación de los cuidadores sobre el cuidado diario, el autocuidado, el manejo de muñones, la coordinación de la atención y la derivación a recursos comunitarios, asegurando así que los cuidadores se sientan empoderados y apoyados.

Para mejorar el valor práctico de estos hallazgos, es crucial detallar su implementación y evaluación. Los proveedores de atención médica y los responsables políticos pueden integrar estas intervenciones en los modelos de atención estandarizando los procesos de derivación, incorporando la educación de los cuidadores en la planificación del alta y asegurando los recursos comunitarios a través de asociaciones formales. La retroalimentación regular de los cuidadores puede utilizarse para evaluar la eficacia de la formación, el apoyo psicológico y los grupos de pares, lo que permite mejoras continuas. Unos pasos claros para la integración y la evaluación reforzarán las aplicaciones en el mundo real de las iniciativas de empoderamiento de los cuidadores familiares.

## CONCLUSIÓN

Los cuidadores familiares desempeñan un papel crucial en el apoyo a las personas amputadas, proporcionando cuidados tanto emocionales como físicos, al tiempo que gestionan importantes cambios en el estilo de vida y desafíos emocionales. A menudo se enfrentan al aislamiento, al miedo y a problemas de salud mental, lo que subraya la necesidad de dar prioridad a su bienestar. Empoderar a los cuidadores requiere un enfoque integral, que incluya recursos educativos, capacitación en habilidades, servicios de apoyo a domicilio y acceso a apoyo social y psicológico. Fortalecer las redes sociales y abordar el bienestar emocional son esenciales para mejorar la calidad de vida tanto de los cuidadores como de los amputados.

Esta investigación destaca el profundo impacto de la amputación de miembros inferiores de causa vascular en los cuidadores y la necesidad crítica de sistemas de apoyo integrales para abordar sus necesidades cambiantes durante la transición del hospital al hogar. Los hallazgos enfatizan la importancia de desarrollar programas e intervenciones de empoderamiento centrados en la familia que integren estrategias hospitalarias, como la capacitación de cuidadores y el desarrollo de habilidades para las tareas de cuidado diario y autocuidado, con intervenciones comunitarias, que incluyen apoyo financiero, redes de pares y atención de salud mental.

Las futuras investigaciones deberían explorar la implementación y la eficacia de estos programas para evaluar su potencial para reducir la carga de los cuidadores, fomentar el empoderamiento y mejorar los resultados tanto para los cuidadores como para los amputados.

## Data Availability

Los datos de este estudio están disponibles previa solicitud al autor de correspondencia.
